# Extensive transcriptomic study emphasizes importance of vesicular transport in *C9orf72* expansion carriers

**DOI:** 10.1186/s40478-019-0797-0

**Published:** 2019-10-08

**Authors:** Dennis W. Dickson, Matthew C. Baker, Jazmyne L. Jackson, Mariely DeJesus-Hernandez, NiCole A. Finch, Shulan Tian, Michael G. Heckman, Cyril Pottier, Tania F. Gendron, Melissa E. Murray, Yingxue Ren, Joseph S. Reddy, Neill R. Graff-Radford, Bradley F. Boeve, Ronald C. Petersen, David S. Knopman, Keith A. Josephs, Leonard Petrucelli, Björn Oskarsson, John W. Sheppard, Yan W. Asmann, Rosa Rademakers, Marka van Blitterswijk

**Affiliations:** 1 Department of Neuroscience, Mayo Clinic, 4500 San Pablo Road, Jacksonville, FL 32224 USA; 2 Department of Health Sciences Research, Mayo Clinic, 200 1st St SW, Rochester, MN 55905 USA; 3 Division of Biomedical Statistics and Informatics, Mayo Clinic, 4500 San Pablo Road, Jacksonville, FL 32224 USA; 4 Department of Health Sciences Research, Mayo Clinic, 4500 San Pablo Road, Jacksonville, FL 32224 USA; 5 Department of Neurology, Mayo Clinic, 4500 San Pablo Road, Jacksonville, FL 32224 USA; 6 Department of Neurology, Mayo Clinic, 200 1st St SW, Rochester, MN 55905 USA; 7 Gianforte School of Computing, Montana State University, 357 Barnard Hall, Bozeman, MT 59717 USA

**Keywords:** Frontotemporal dementia, Frontotemporal lobar degeneration, Amyotrophic lateral sclerosis, Motor neuron disease, C9orf72, Transcriptomics, Vesicular transport, Machine learning, RNA sequencing, Repeat expansion disorders

## Abstract

The majority of the clinico-pathological variability observed in patients harboring a repeat expansion in the C9orf72-SMCR8 complex subunit (*C9orf72*) remains unexplained. This expansion, which represents the most common genetic cause of frontotemporal lobar degeneration (FTLD) and motor neuron disease (MND), results in a loss of *C9orf72* expression and the generation of RNA foci and dipeptide repeat (DPR) proteins. The C9orf72 protein itself plays a role in vesicular transport, serving as a guanine nucleotide exchange factor that regulates GTPases. To further elucidate the mechanisms underlying *C9orf72*-related diseases and to identify potential disease modifiers, we performed an extensive RNA sequencing study. We included individuals for whom frontal cortex tissue was available: FTLD and FTLD/MND patients with (*n* = 34) or without (*n* = 44) an expanded *C9orf72* repeat as well as control subjects (*n* = 24). In total, 6706 genes were differentially expressed between these groups (false discovery rate [FDR] < 0.05). The top gene was *C9orf72* (FDR = 1.41E-14), which was roughly two-fold lower in *C9orf72* expansion carriers than in (disease) controls. Co-expression analysis revealed groups of correlated genes (modules) that were enriched for processes such as protein folding, RNA splicing, synaptic signaling, metabolism, and Golgi vesicle transport. Within our cohort of *C9orf72* expansion carriers, machine learning uncovered interesting candidates associated with clinico-pathological features, including age at onset (vascular endothelial growth factor A [*VEGFA*]), *C9orf72* expansion size (cyclin dependent kinase like 1 [*CDKL1*]), DPR protein levels (eukaryotic elongation factor 2 kinase [*EEF2K*]), and survival after onset (small G protein signaling modulator 3 [*SGSM3*]). Given the fact that we detected a module involved in vesicular transport in addition to a GTPase activator (*SGSM3*) as a potential modifier, our findings seem to suggest that the presence of a *C9orf72* repeat expansion might hamper vesicular transport and that genes affecting this process may modify the phenotype of *C9orf72*-linked diseases.

**Electronic supplementary material:**

The online version of this article (10.1186/s40478-019-0797-0) contains supplementary material, which is available to authorized users.

## Introduction

Substantial clinical and pathological variability has been reported in patients carrying an expanded repeat in the C9orf72-SMCR8 complex subunit (*C9orf72*) [58], which leads to frontotemporal dementia (FTD) and amyotrophic lateral sclerosis (ALS) [14, 50]. While FTD is the second most frequent cause of dementia in the presenile group, ALS is the most common form of motor neuron disease (MND). Intriguingly, there is considerable clinical, genetic, and pathological overlap between FTD and ALS. In fact, up to 40% of FTD patients demonstrate motor neuron involvement [7, 44]. Similarly, up to 50% of ALS patients have cognitive impairment and 15% fulfill the FTD criteria [17, 46]. Mutations in several genes appear to be specific for either FTD or ALS (e.g., superoxide dismutase 1 [*SOD1*]); however, most have been detected in both diseases, like the repeat expansion in *C9orf72*. Furthermore, TAR DNA-binding protein 43 (TDP-43) inclusions can be observed in approximately 50% of FTD patients and more than 90% of ALS patients [43, 44]. Given this overlap, FTD and ALS are thought to represent a disease spectrum.

The repeat expansion in *C9orf72* accounts for about 30% of familial cases and 5–10% of sporadic cases [41, 58], possibly due to a reduction in *C9orf72* expression [14], the aggregation of flawed RNA transcripts in the nucleus of cells (RNA foci) [14], and the formation of repetitive proteins aberrantly translated from the expansion (dipeptide repeat [DPR] proteins) [4, 42]. The C9orf72 protein itself is known to interact with endosomes and functions in vesicle trafficking [18, 56].

Thus far, a limited number of studies has been performed to investigate the expression pattern of *C9orf72*-linked diseases. We have, for instance, profiled brain tissue of *C9orf72* expansion carriers using expression arrays, which uncovered an upregulation of transthyretin and homeobox genes [19]. In an RNA sequencing study, we also examined differential expression, alternative splicing, and alternative polyadenylation in ALS patients harboring a *C9orf72* expansion [47]. We detected widespread transcriptome changes in the cerebellum, particularly of RNA-processing events [47]. Furthermore, we observed elevated levels of repetitive elements (e.g., long interspersed nuclear elements [LINEs]) in patients with a *C9orf72* repeat expansion [48]. Several other studies also investigated expression patterns distinctive of an expanded repeat in *C9orf72* by examination of laser-captured motor neurons, lymphoblastoid cell lines, fibroblast and induced pluripotent stem cell (iPSC) lines, iPSC-derived motor neuron cultures, and/or postmortem motor cortex tissue from *C9orf72* expansion carriers [11, 16, 30, 52, 54].

Despite these efforts, the majority of the clinico-pathological variability remains unexplained in *C9orf72* expansion carriers. As such, we have performed an in-depth RNA sequencing study on frontal cortex tissue from a well-characterized cohort. We evaluated individuals who received a pathological diagnosis of frontotemporal lobar degeneration (FTLD) with or without MND as well as control subjects stored at the Mayo Clinic Florida Brain Bank (*n* = 102). In addition to differential expression and co-expression analyses, we used various analytical approaches within the group of *C9orf72* expansion carriers to identify genes associated with clinical and pathological features of *C9orf72*-related diseases. Our findings provide additional evidence for the involvement of vesicle-mediated transport and reveal several potential modifiers of *C9orf72*-linked diseases.

## Materials and methods

### Subjects

Subjects were selected for whom frozen brain tissue was available in our Mayo Clinic Florida Brain Bank (*n* = 102; Table 1). Frontal cortex tissue was collected from the middle frontal gyrus at the level of the nucleus accumbens. We included *C9orf72* expansion carriers (*n* = 34) pathologically diagnosed with FTLD characterized by TDP-43 inclusions (FTLD-TDP) in the presence or absence of MND, patients with FTLD-TDP or FTLD/MND without known mutations (type A or B; *n* = 44), and control subjects without neurological diseases (*n* = 24). Our *C9orf72* expansion carriers had a median age at death of 69 years (interquartile range [IQR]: 62–76), a median RNA integrity number (RIN) of 8.9 (IQR: 8.4–9.5), and 35% was female. For patients without a repeat expansion, the median age at death was 78 years (IQR: 68–83), their median RIN value was 9.6 (IQR: 9.1–9.8), and 50% was female. The median age at death of control subjects was 87 years (IQR: 78–89) with a median RIN value of 9.1 (IQR: 8.8–9.6) and 67% was female. Of note, in previous studies, we already obtained the expansion size, RNA foci burden, and DPR protein levels for the majority of our expansion carriers [13, 21, 57]. Methylation levels of the *C9orf72* promoter were determined using 100 ng of DNA as input material with a quantitative methylation-sensitive restriction enzyme-based assay, as described elsewhere [40, 51].

**Table 1:** Subject characteristics

Variable	C9Plus (*n* = 34)	C9Minus (*n* = 44)	Control (*n* = 24)
Age at death (years)	69.0 (62.0–75.8)	78.0 (67.8–83.2)	86.5 (78.2–89.2)
RIN (value)	8.9 (8.4–9.5)	9.6 (9.1–9.8)	9.1 (8.8–9.6)
Sex (female)	12 (35%)	22 (50%)	16 (67%)
Diagnosis (FTLD/MND)	12 (35%)	13 (30%)	0 (0%)

Data are sample median (interquartile range [IQR]) or number (%). Information is shown for patients carrying a *C9orf72* repeat expansion (C9Plus), patients without this repeat expansion (C9Minus), and control subjects without neurological diseases (Control). Age at death, RNA integrity number (RIN), sex, and pathological diagnosis (frontotemporal lobar degeneration [FTLD] with motor neuron disease [MND]) are specified

### RNA sequencing

Total RNA was extracted from frozen brain tissue using the RNeasy Plus Mini Kit (Qiagen). RNA quality and quantity were determined with a 2100 Bioanalyzer Instrument (Agilent) using the RNA Nano Chip (Agilent); only samples with a RIN value above 7.0 were included. Libraries were made using the TruSeq RNA Library Prep Kit (Illumina; v2) and sequenced at 10 samples/lane as paired-end 101 base-pair reads on a HiSeq 4000 (Illumina) at Mayo Clinic’s Genome Analysis Core. Subsequently, raw sequencing reads were aligned to the human reference genome (GRCh38) with Spliced Transcripts Alignment to a Reference (STAR; v2.5.2b) [15]. After alignment, library quality was assessed using RSeQC (v3.0.0) [60], and gene-level expression was quantified using the Subread package (v1.5.1) [37]. All analyses described below were performed in R (R Core Team; v3.5.3).

### Differential expression analysis

We used conditional quantile normalization (CQN) to account for differences in gene counts, gene lengths, and GC content, resulting in comparable quantile-by-quantile distributions across samples [24, 49]. Genes were kept if their maximum normalized and log2-transformed reads per kb per million (RPKM) values were above zero (*n* = 24,092). Using linear regression models, source of variation (SOV) analysis was then performed to determine how much variation was explained by the disease group (*C9orf72* expansion carriers, non-expansion carriers, and controls) as well as by potential confounders (RIN, sex, age at death, plate, and gene counts). We also assessed the effects of differences in cellular composition between individuals using surrogate markers for five major cell types: neurons (enolase 2 [*ENO2*]), microglia (CD68 molecule [*CD68*]), astrocytes (glial fibrillary acidic protein [*GFAP*]), oligodendrocytes (oligodendrocyte transcription factor 2 [*OLIG2*]), and endothelial cells (CD34 molecule [*CD34*]) [1, 12, 23]. Based on our SOV analysis, variables with a mean F-statistic above 1.25 were selected. Differential expression analysis was performed using two separate linear regression models: one model included RIN, sex, age at death, plate, and disease group, while the other model also included our five surrogate markers for the major cell types. Fold-changes were determined and *p*-values were adjusted for multiple testing using a false discovery rate (FDR) procedure [5]. Genes with an FDR below 5% were considered statistically significant (FDR < 0.05). To examine whether significantly differentially expressed genes were enriched for biological processes and pathways, enrichment analysis was performed using the anRichment package [33] and gene sets from the molecular signatures database (MSigDB; v6.2) [39]. For visualization purposes, Venn diagrams were generated with the VennDiagram package [10]. Moreover, heat maps were made with the ComplexHeatmap package [22] and the flashClust package [35], utilizing the Euclidean distance and average method.

### Co-expression analysis

In addition to the gene-level analyses described in the previous section, we performed module-level analyses to identify the building blocks of biological systems, revealing relevant information about the system’s structure and dynamics as well as the function of certain proteins [61]. As such, we employed weighted gene co-expression network analysis (WGCNA) to find modules comprised of highly correlated genes that go up or down together [34], using residual expression values adjusted for aforementioned potential confounders as input (both with and without surrogate markers). Separate analyses were performed for each pairwise comparison, creating signed hybrid networks and using the biweight midcorrelation (bicor) method. To achieve a scale-free topology, we selected a power appropriate for each comparison, ranging between 4 and 14. A dynamic tree cutting method was used with a minimum module size of 30 and a merge height varying from 0.25 to 0.35, depending on the comparison. Modules generated using these settings were represented by their first principal component (module eigengene) and a unique color. For every gene, we calculated correlations between expression levels and each module’s eigengene value (module membership). Modules that differed significantly between disease groups were further investigated using enrichment analyses and displayed with heat maps, using methods identical to those described above. Additionally, network visualization was performed for top protein-coding genes belonging to modules of interest with a relatively high module membership (> 0.6), utilizing the force-directed yFiles Organic Layout and Organic Edge Router algorithms in Cytoscape (v3.7.1) [55]. In these network plots, the connectivity of each gene was represented by the size of its node, the module to which it has been assigned by its color, and the strength of the correlation by the thickness of its edges.

### Clinico-pathological association analysis

To find associations with clinical and pathological features of the disease in patients carrying an expanded *C9orf72* repeat (*n* = 34), we obtained residuals from linear regression models with expression levels as outcome to account for potential confounders (RIN, sex, and plate, either with or without surrogate markers). First, we performed analyses to examine individual genes, starting with linear regression models. We investigated associations with age at onset and age at death, adjusting for disease subgroup (FTLD or FTLD/MND). Subsequently, we assessed associations with *C9orf72* expansion size, RNA foci burden (mean percentage of cells with sense or antisense RNA foci), DPR protein levels (total poly[GP]), and methylation of the *C9orf72* promoter, while adjusting for disease subgroup and age at death. Hereafter, we performed a logistic regression analysis to compare expression levels between patients with predominant FTLD to those diagnosed with both FTLD and MND, adjusting for age at death. We ran Cox proportional hazard regression models, including disease subgroup and age at death as potential confounders. Hazard ratios (HRs) and 95% confidence intervals (CIs) were estimated; deaths of any cause were utilized as our survival endpoint. Three approaches were used for our survival analysis to assess expression levels: comparing the top 50% to the bottom 50% as a dichotomous categorical variable, ranking expression levels from low to high, and examining them as a continuous variable. Notably, all models were adjusted for multiple testing using an FDR procedure [5]; an FDR below 5% was considered statistically significant (FDR < 0.05).

Second, we evaluated combinations of genes found to be nominally significant in our single-gene analysis (*P* < 0.05). To examine the sensitivity of our results, we opted to use two machine learning methods, namely Least Absolute Shrinkage and Selection Operator (LASSO) regression and random forest. LASSO regression was performed with the glmnet package [20]. The most parsimonious model was selected, using leave-one-out cross-validation, an alpha of one, and a lambda within one standard error from the model with the lowest cross-validation error (mean squared error, classification error, or partial-likelihood deviance). This approach was employed using models appropriate for the nature of the given response variable, including age at onset, age at death, expansion size, RNA foci burden, poly(GP) DPR levels, *C9orf72* promoter methylation, disease subgroup, and survival after onset. We then used the randomForest package [38], which implements Breiman’s random forest algorithm [6]. We tuned the number of trees in the forest (1000 to 30,000), the number of features considered at each split (2 to 98), and the size of terminal nodes (2 to 10). Subsequently, we created a random forest regressor (age at onset, age at death, *C9orf72* expansion size, RNA foci levels, DPR proteins, and promoter methylation) or classifier (disease subgroup). We extracted the out-of-bag error rate as well as information about the importance of each gene (variable importance), as represented by its permuted effect on the error rate (e.g., mean squared error or accuracy), while other genes remained unchanged [38].

### Validation experiments and analysis

We validated RNA expression levels of the top candidate genes in *C9orf72* expansion carriers from our RNA sequencing cohort (*n* = 34). Reverse transcription was performed using 250 ng of RNA as template with the SuperScript III Kit (Invitrogen) and an equal ratio of Random Hexamers and Oligo dT primers. The following expression assays (TaqMan) were performed: vascular endothelial growth factor A (*VEGFA*; Hs00900055_m1), cyclin dependent kinase like 1 (*CDKL1*; Hs01012519_m1), eukaryotic elongation factor 2 kinase (*EEF2K*; Hs00179434_m1), and small G protein signaling modulator 3 (*SGSM3*; Hs00924186_g1). As markers, *ENO2* (Hs00157360_m1) and *GFAP* (Hs00909233_m1) were selected. To obtain relative expression levels for each patient, the median of replicates was taken, the geometric mean of the two markers was calculated, and a calibrator on every plate was used for normalization, utilizing the ΔΔCt method. Subsequently, the correlation between these relative expression levels and residuals from our RNA sequencing analysis was calculated using a Spearman’s test of correlation.

## Results

### Top differentially expressed gene is C9orf72

**Fig. 1:**
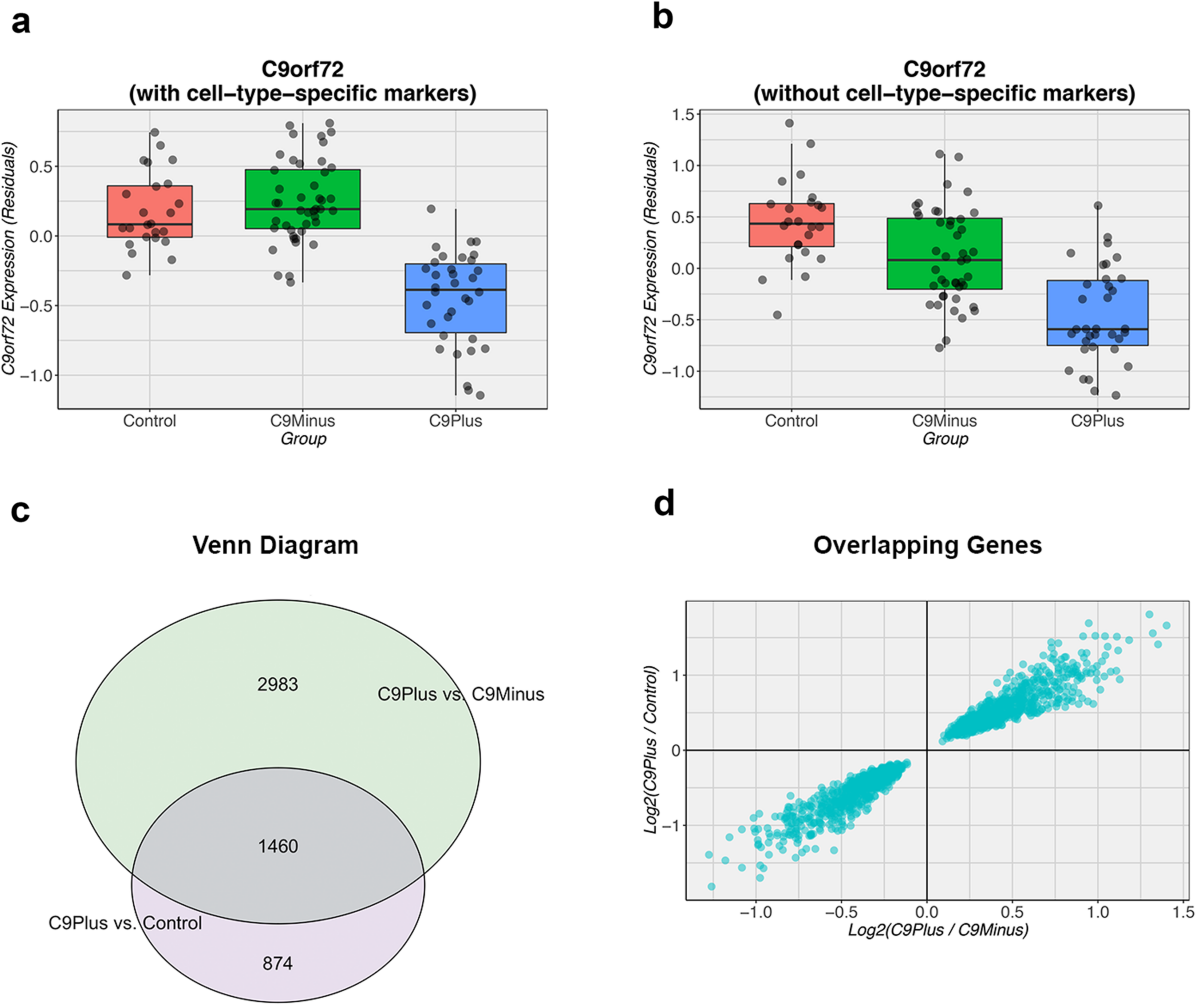
**a** After adjustment for five major cell types (neurons, microglia, astrocytes, oligodendrocytes, and endothelial cells), expression levels of *C9orf72* are shown for all disease groups: patients with a *C9orf72* repeat expansion (C9Plus), patients without this expansion (C9Minus), and control subjects (Control). **b** Without adjustment for five cell types, the expression levels of *C9orf72* are displayed for C9Plus, C9Minus, and Control. Importantly, in both graphs, *C9orf72* levels are lower in C9Plus than in C9Minus or Control. For each box plot, the median is represented by a *solid black line*, and each box spans the interquartile range (IQR; 25th percentile to 75th percentile). **c** In total, 4443 differentially expressed genes are detected when comparing C9Plus to C9Minus. The comparison between C9Plus and Control results in 2334 differentially expressed genes. As displayed in the Venn diagram, 1460 differentially expressed genes overlap. **d** All overlapping genes go in the same direction (lower left quadrant and upper right quadrant)

**Fig. 2:**
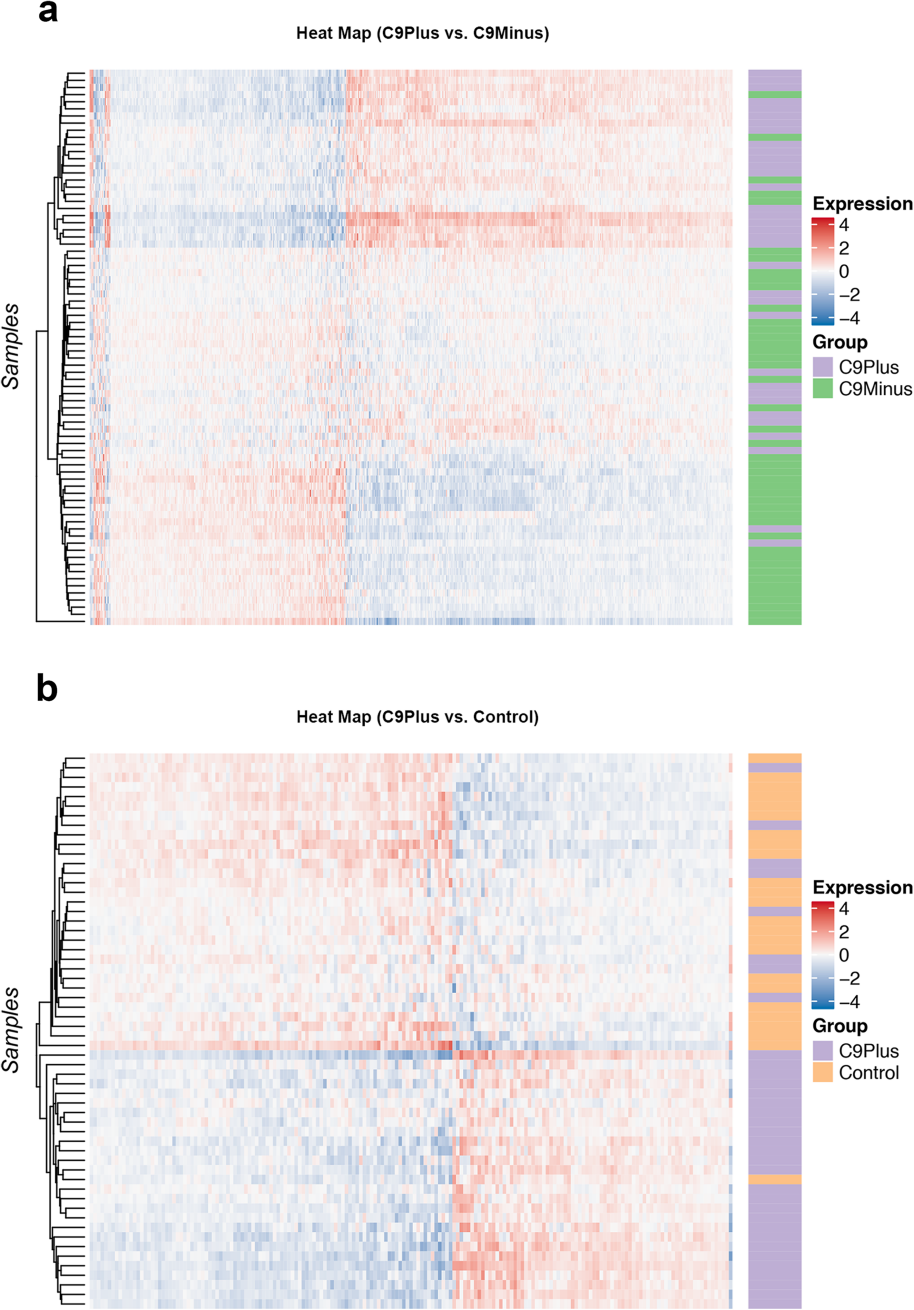
**a** When comparing patients with a *C9orf72* repeat expansion to those without this expansion (C9Plus vs. C9Minus), a heat map is displayed. **b** A heat map is shown when comparing expansion carriers to control subjects (C9Plus vs. Control). In these heat maps, high expression levels are shown in *red* and low levels in *blue*. Both heat maps indicate that most expansion carriers cluster together (*purple*). Of note, for visualization purposes, only the top differentially expressed genes are displayed (false discovery rate [FDR] < 0.001)

We performed RNA sequencing on carriers of a *C9orf72* repeat expansion (n = 34), FTLD and FTLD/MND patients without this expansion (*n* = 44), and control subjects without any neurological disease (*n* = 24; Table 1). When adjusting for cell-type-specific markers, 6706 genes were significantly different between these groups. Without adjustment, 11,770 genes were differentially expressed. Importantly, the top gene was *C9orf72* itself, both with (FDR = 1.41E-14) and without (FDR = 8.69E-08) adjustment for cell-type-specific markers (Table 2; Fig. 1a, b). Hereafter, we specifically compared patients with a *C9orf72* expansion to patients without this expansion or to controls. For simplicity, we focused on results that accounted for differences in cellular composition. In total, we detected 4443 differentially expressed genes when comparing expansion carriers to patients without this expansion and 2334 genes when comparing them to controls (Fig. 1c). Heat maps demonstrated that most patients with an expanded repeat clustered together (Fig. 2), especially when comparing them to controls. Of the differentially expressed genes, 1460 overlapped (Fig. 1c, d), including *C9orf72* itself. The RNA expression levels of *C9orf72* were roughly two-fold lower in expansion carriers than in non-expansion carriers (FDR = 6.04E-06) or control subjects (FDR = 1.08E-05; Table 3). We further investigated overlapping genes using enrichment analyses, which indicated that these genes might be enriched for processes involved in endocytosis (FDR = 0.02; Table 4).

**Table 2:** Differential Expression (All Groups)

Order	With Cell-Type-Specific Markers				Without Cell-Type-Specific Markers			
	Gene	Chr	*P*-value	FDR	Gene	Chr	*P*-value	FDR
1	*C9orf72*	chr9	5.86E-19	1.41E-14	*C9orf72*	chr9	3.61E-12	8.69E-08
2	*SMIM14*	chr4	2.06E-11	2.49E-07	*RP11-196E1.3*	chr11	3.89E-11	4.68E-07
3	*FBLL1*	chr5	1.12E-10	8.96E-07	*GEM*	chr8	3.45E-10	2.05E-06
4	*ABCE1*	chr4	3.54E-10	2.13E-06	*PCP4*	chr21	3.47E-10	2.05E-06
5	*HRH3*	chr20	9.60E-10	4.62E-06	*KCNC3*	chr19	5.85E-10	2.05E-06
6	*GIT1*	chr17	1.41E-09	5.55E-06	*ANGPT1*	chr8	6.33E-10	2.05E-06
7	*AC009133.15*	chr16	1.61E-09	5.55E-06	*SMIM14*	chr4	6.85E-10	2.05E-06
8	*PYCR2*	chr1	2.33E-09	6.24E-06	*GPCPD1*	chr20	7.04E-10	2.05E-06
9	*VSTM2A-OT1*	chr7	2.52E-09	6.24E-06	*KREMEN2*	chr16	8.08E-10	2.05E-06
10	*MTR*	chr1	2.59E-09	6.24E-06	*DPYSL3*	chr5	8.49E-10	2.05E-06
11	*KCNJ6*	chr21	3.06E-09	6.26E-06	*SCN1B*	chr19	9.75E-10	2.14E-06
12	*RP11-196E1.3*	chr11	3.26E-09	6.26E-06	*PRELP*	chr1	1.38E-09	2.78E-06
13	*TESK1*	chr9	3.98E-09	6.26E-06	*FBLL1*	chr5	1.58E-09	2.93E-06
14	*DCAF16*	chr4	4.07E-09	6.26E-06	*LINC01102*	chr2	1.95E-09	3.17E-06
15	*SH3GL1P3*	chr17	5.26E-09	6.26E-06	*CTD-2126E3.4*	chr19	1.98E-09	3.17E-06
16	*DCTN6*	chr8	5.26E-09	6.26E-06	*SLC17A6*	chr11	2.10E-09	3.17E-06
17	*BPGM*	chr7	5.57E-09	6.26E-06	*RAB4B*	chr19	2.23E-09	3.17E-06
18	*NT5DC1*	chr6	5.58E-09	6.26E-06	*KB-1107E3.1*	chr8	3.56E-09	4.77E-06
19	*RP11-318A15.7*	chr17	5.59E-09	6.26E-06	*CCDC102A*	chr16	4.10E-09	5.19E-06
20	*CHMP2B*	chr3	5.69E-09	6.26E-06	*AEBP1*	chr7	4.62E-09	5.39E-06

Differentially expressed genes are displayed either with or without adjustment for cell-type-specific markers. For each gene, the chromosome (Chr), *p*-value, and false discovery rate (FDR) are included

**Table 3:** Differential Expression (Specific Comparisons)

Order	C9Plus vs. C9Minus					C9Plus vs. Control				
	Gene	Chr	*P*-value	FDR	FC	Gene	Chr	*P*-value	FDR	FC
1	*C9orf72*	chr9	4.35E-10	6.04E-06	-1.75	*C9orf72*	chr9	4.48E-10	1.08E-05	-1.69
2	*FBLL1*	chr5	5.01E-10	6.04E-06	1.88	*SMIM14*	chr4	3.16E-09	3.80E-05	-1.34
3	*VSTM2A-OT1*	chr7	1.51E-09	9.64E-06	1.96	*PYCR2*	chr1	5.96E-09	3.99E-05	-1.73
4	*KCNJ6*	chr21	1.88E-09	9.64E-06	1.92	*NT5DC1*	chr6	9.62E-09	3.99E-05	-1.57
5	*ABCE1*	chr4	2.06E-09	9.64E-06	-1.35	*GID4*	chr17	1.08E-08	3.99E-05	1.33
6	*SMIM14*	chr4	2.40E-09	9.64E-06	-1.25	*GIT1*	chr17	1.10E-08	3.99E-05	1.63
7	*HRH3*	chr20	4.12E-09	1.40E-05	1.99	*SCN3A*	chr2	1.16E-08	3.99E-05	1.94
8	*SH3GL1P3*	chr17	4.65E-09	1.40E-05	1.80	*S100B*	chr21	1.38E-08	4.17E-05	-1.94
9	*CTD-2126E3.4*	chr19	5.61E-09	1.50E-05	2.11	*CMTM5*	chr14	1.71E-08	4.57E-05	-2.49
10	*LRFN4*	chr11	7.15E-09	1.72E-05	1.69	*RP11-196E1.3*	chr11	1.96E-08	4.72E-05	-2.90
11	*RP11-147 L13.13*	chr17	1.02E-08	2.24E-05	1.80	*DCTN6*	chr8	3.88E-08	6.82E-05	-1.33
12	*CA7*	chr16	1.14E-08	2.28E-05	1.69	*SUCLG2*	chr3	4.11E-08	6.82E-05	-1.72
13	*MXD4*	chr4	1.36E-08	2.47E-05	1.35	*MARCKSL1*	chr1	4.16E-08	6.82E-05	-1.57
14	*STX12*	chr1	1.43E-08	2.47E-05	-1.23	*KCNG1*	chr20	4.17E-08	6.82E-05	2.88
15	*ATG4D*	chr19	1.73E-08	2.78E-05	1.30	*BAG2*	chr6	4.25E-08	6.82E-05	1.47
16	*DUSP14*	chr17	1.84E-08	2.78E-05	1.34	*RAF1*	chr3	5.09E-08	7.67E-05	1.23
17	*CHMP2B*	chr3	1.97E-08	2.78E-05	-1.32	*TESK1*	chr9	5.57E-08	7.81E-05	1.33
18	*LPHN1*	chr19	2.08E-08	2.78E-05	1.43	*BDNF-AS*	chr11	6.20E-08	7.81E-05	-1.82
19	*ST8SIA5*	chr18	2.31E-08	2.93E-05	1.52	*CTC-273B12.10*	chr19	6.57E-08	7.81E-05	2.69
20	*NFATC2IP*	chr16	2.55E-08	3.07E-05	-1.29	*CYP4F11*	chr19	6.61E-08	7.81E-05	-3.25

Differentially expressed genes are shown when comparing patients with an expanded *C9orf72* repeat to those without this repeat (C9Plus vs. C9Minus) or to control subjects (C9Plus vs. Control). For each gene, the chromosome (Chr), *p*-value, false discovery rate (FDR), and fold-change (FC) are displayed. Of note, this table has been generated after adjustment for cell-type-specific markers

**Table 4:** Enrichment Analysis (Overlapping Genes)

Source	Order	Process/Pathway	*P*-value	FDR
KEGG	1	KEGG_ENDOCYTOSIS	4.40E-05	0.02
	2	KEGG_CITRATE_CYCLE_TCA_CYCLE	0.004	0.38
GO-BP	1	GO_ORGANIC_ACID_METABOLIC_PROCESS	7.91E-05	0.27
	2	GO_TRICARBOXYLIC_ACID_METABOLIC_PROCESS	1.40E-04	0.27

Results of enrichment analyses are shown for overlapping genes. For each process or pathway, the *p*-value and false discovery rate (FDR) are included. Enrichment analyses were performed using Kyoto Encyclopedia of Genes and Genomes (KEGG) and Gene Ontology – Biological Processes (GO-BP). Of note, this table has been generated after adjustment for cell-type-specific markers

### Co-expression analysis reveals relevant modules involved in processes like vesicular transport

**Fig. 3:**
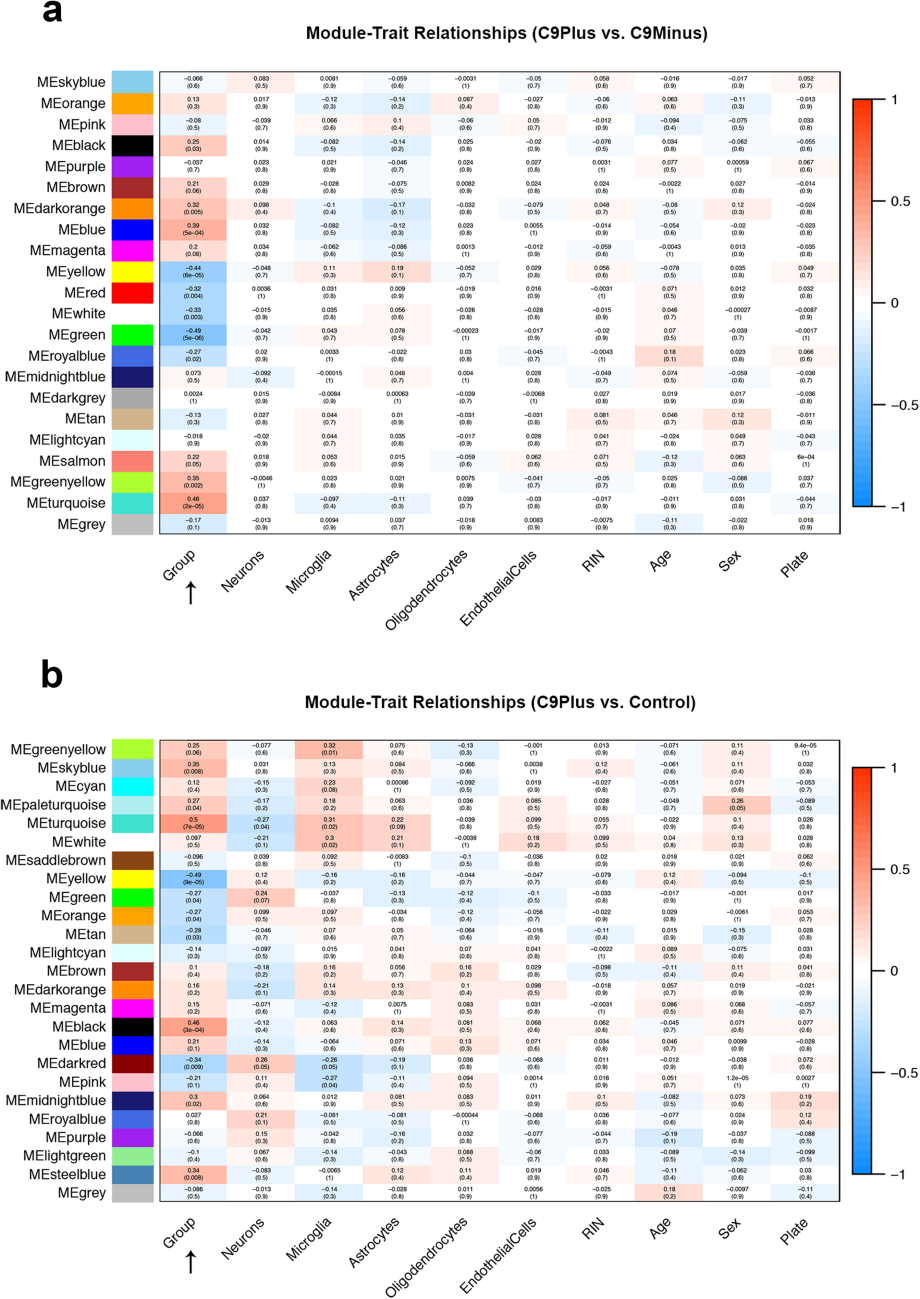
**a** Module-trait relationships are presented for patients with an expanded *C9orf72* repeat and patients without this repeat (C9Plus vs. C9Minus). **b** For patients with an expansion and control subjects (C9Plus vs. Control), module-trait relationships are plotted. These plots are generated with weighted gene co-expression network analysis (WGCNA) to find groups of genes that go up (*red*) or down (*blue*) together. A unique color has been assigned to each of these groups, also called a module. Correlations and *p*-values are shown for variables of interest, including disease group (C9Plus, C9Minus, and/or Control; *arrow*), neurons, microglia, astrocytes, oligodendrocytes, endothelial cells, RNA integrity number (RIN), age at death, sex, and plate. The strongest correlations (*brightest colors*) are observed for the disease group. Notably, both module-trait relationship plots are based on residuals obtained after adjustment for cell-type-specific markers

**Fig. 4:**
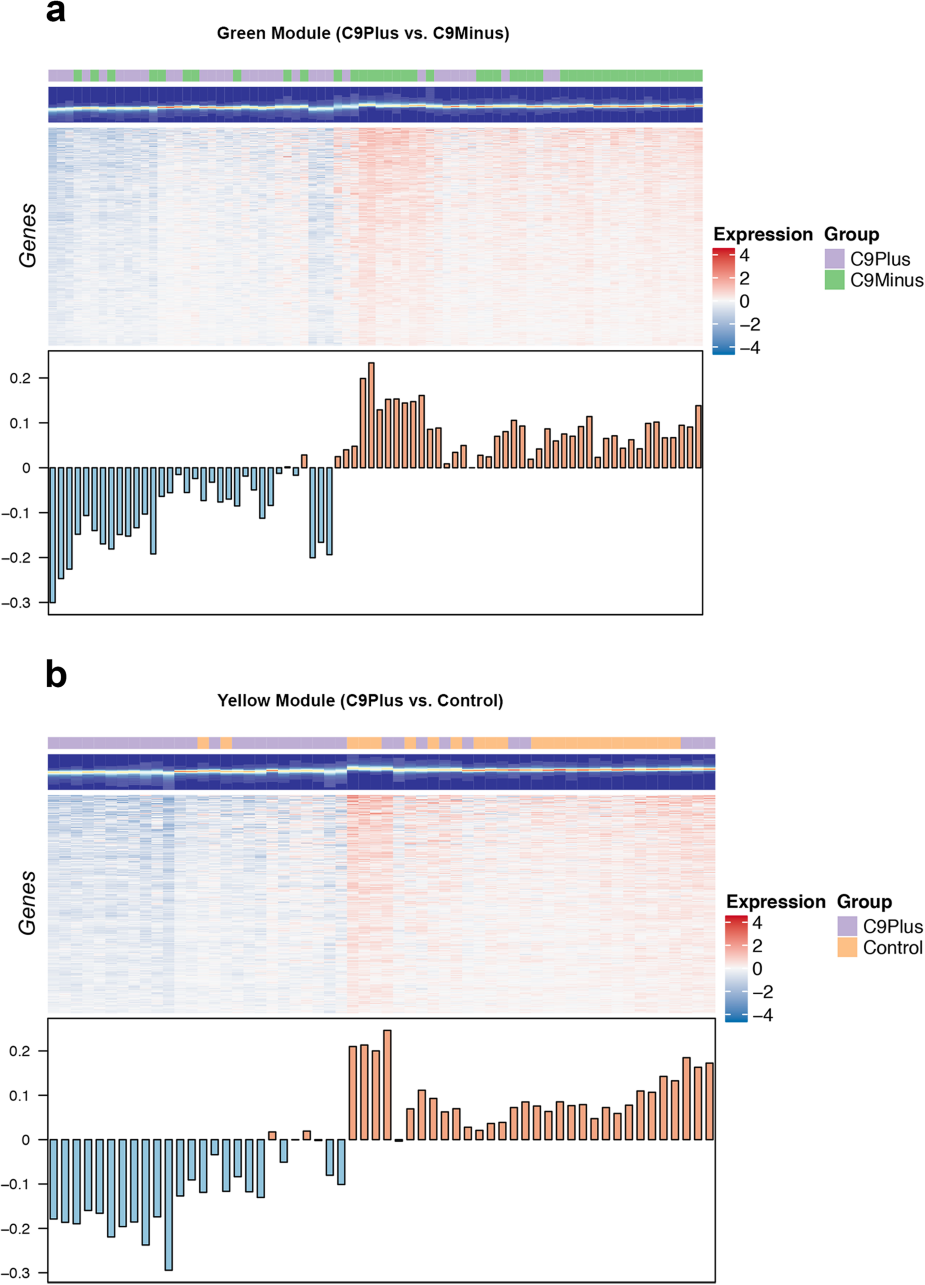
**a** One specific group of genes is visualized in a heat map: the green module. **b** A heat map is displayed for the yellow module. High expression levels are shown in *red* and low levels in *blue*. Below every heat map, the first principal component of a given module (module eigengene) is displayed for each sample. Most *C9orf72* expansion carriers (C9Plus) appear to have relatively low levels as compared to patients without this expansion (C9Minus) or to control subjects (Control)

**Fig. 5:**
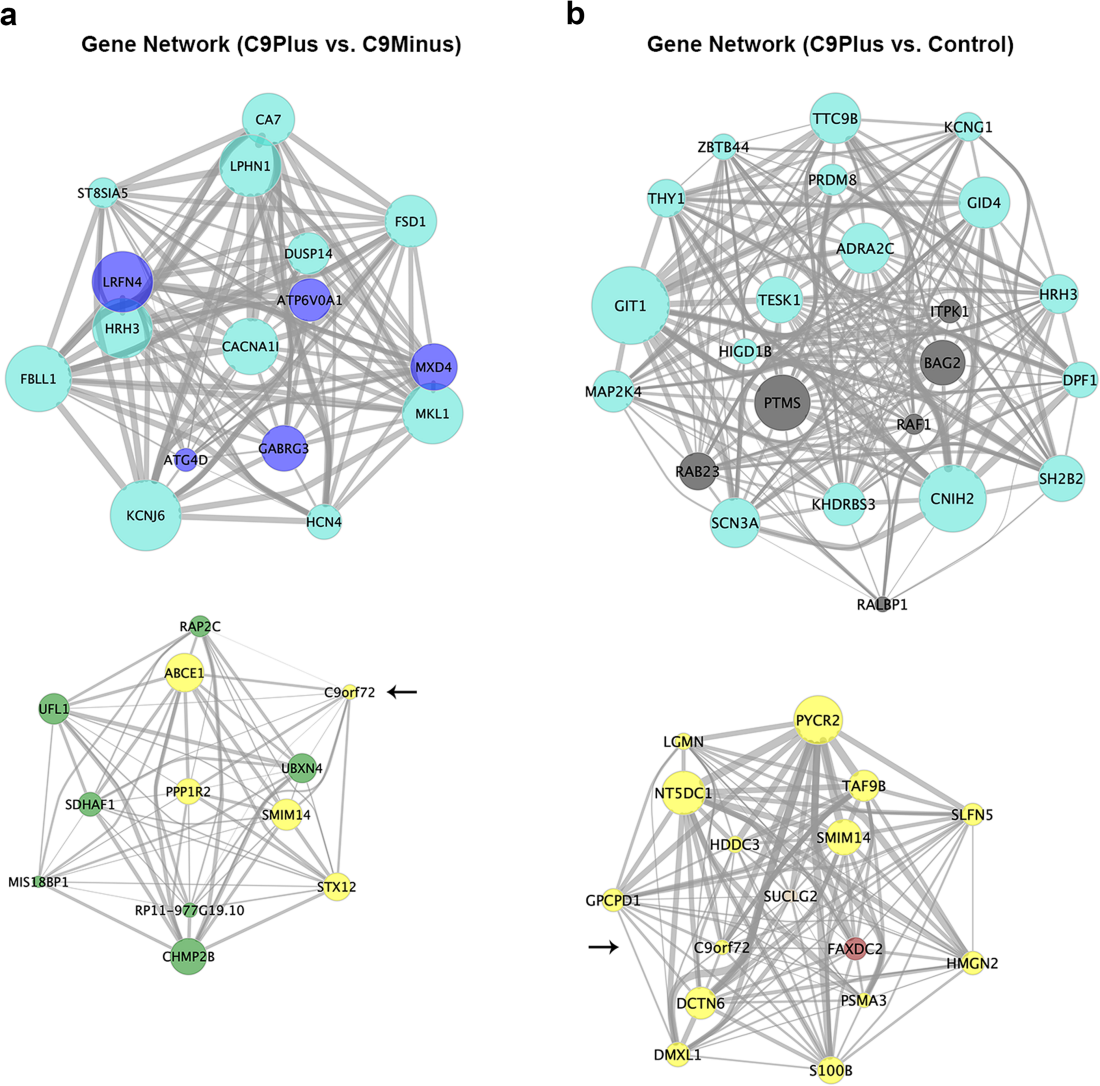
**a** For patients harboring a *C9orf72* repeat expansion and those without this expansion (C9Plus vs. C9Minus; module membership > 0.6 and significance < 1.0E-06), a gene network is displayed. **b** A gene network is visualized when examining expansion carriers and controls (C9Plus vs. Control; module membership > 0.6 and significance < 2.5E-05). In these network plots, the connectivity of each gene is represented by the *size* of its node, the module to which it has been assigned by its *color*, and the strength of the correlation by the *thickness* of its edges; the *C9orf72* gene is denoted by an *arrow*. Of note, the plots in this figure have been generated after adjustment for cell-type-specific markers

Next, we performed module-level analyses using WGCNA. When comparing patients with an expanded *C9orf72* repeat to those without this repeat, we identified 22 modules. Visualization of the module-trait relationships (Fig. 3a), revealed that the strongest relationships were dependent on the presence or absence of a *C9orf72* repeat expansion (disease group). In fact, we only detected significant correlations with the disease group, resulting in the identification of 11 modules of interest. None of these modules demonstrated a significant correlation with potential confounders, such as cellular composition, RIN, age at death, sex, or plate (Fig. 3a). Enrichment analysis of these 11 modules (Table 5) showed that they were involved in protein folding (black), RNA splicing (blue), metabolic processes (yellow), Golgi vesicle transport (green), GABAergic interneuron differentiation (greenyellow), synaptic signaling (turquoise), etc. Given the potential function of the C9orf72 protein, we visualized the green module (Fig. 4a); most expansion carriers appeared to have lower module eigengene values for this module than disease controls. In addition to Golgi vesicle transport (FDR = 1.33E-06), the green module was also significantly enriched for related processes, such as endoplasmic reticulum to Golgi vesicle-mediated transport (FDR = 1.97E-05), vacuolar transport (FDR = 9.91E-05), vesicle-mediated transport (FDR = 0.002), and lysosomes (FDR = 0.002). This is in agreement with the cellular components that appeared to be involved, including vacuolar part (FDR = 4.31E-10), endoplasmic reticulum part (FDR = 2.88E-09), endoplasmic reticulum (FDR = 2.34E-08), vacuole (FDR = 8.41E-08), and vacuolar membrane (FDR = 6.53E-07). A gene network, which displayed top genes from significant modules, demonstrated that members of the green module (e.g., charged multivesicular body protein 2B [*CHMP2B*]) clustered together with genes belonging to the yellow module, most importantly *C9orf72* (Fig. 5a).

**Table 5:** Enrichment Analysis (C9Plus vs. C9Minus)

Module	Dir	Source	Order	Process/Pathway	*P*-value	FDR
MEblack	Up	KEGG	1	KEGG_ANTIGEN_PROCESSING_AND_PRESENTATION	3.31E-06	4.34E-04
			2	KEGG_SPLICEOSOME	3.80E-04	0.02
		GO-BP	1	GO_PROTEIN_FOLDING	4.24E-29	8.97E-26
			2	GO_RESPONSE_TO_TOPOLOGICALLY_INCORRECT_PROTEIN	6.40E-19	7.55E-16
MEdarkorange	Up	KEGG	1	KEGG_ECM_RECEPTOR_INTERACTION	0.04	0.67
			2	KEGG_FOCAL_ADHESION	0.10	1.00
		GO-BP	1	GO_PROTEIN_HETEROOLIGOMERIZATION	0.001	0.06
			2	GO_REGULATION_OF_CARBOHYDRATE_METABOLIC_PROCESS	0.003	0.14
MEblue	Up	KEGG	1	KEGG_SPLICEOSOME	0.002	0.09
			2	KEGG_RNA_DEGRADATION	0.04	0.68
		GO-BP	1	GO_RNA_SPLICING	7.85E-12	4.57E-09
			2	GO_RNA_PROCESSING	9.98E-12	5.56E-09
MEyellow	Down	KEGG	1	KEGG_PEROXISOME	4.48E-06	5.00E-04
			2	KEGG_DNA_REPLICATION	3.65E-04	0.02
		GO-BP	1	GO_SMALL_MOLECULE_METABOLIC_PROCESS	4.33E-09	1.58E-06
			2	GO_CELLULAR_AMINO_ACID_METABOLIC_PROCESS	3.34E-08	1.01E-05
MEred	Down	KEGG	1	KEGG_ECM_RECEPTOR_INTERACTION	1.52E-07	3.38E-05
			2	KEGG_FOCAL_ADHESION	3.67E-06	4.34E-04
		GO-BP	1	GO_EXTRACELLULAR_STRUCTURE_ORGANIZATION	1.56E-16	1.45E-13
			2	GO_TISSUE_DEVELOPMENT	2.61E-12	1.54E-09
MEwhite	Down	KEGG	1	KEGG_RIBOSOME	1.49E-05	0.001
			2	KEGG_PROTEIN_EXPORT	0.01	0.32
		GO-BP	1	GO_PEPTIDE_METABOLIC_PROCESS	1.57E-07	4.08E-05
			2	GO_CELLULAR_AMIDE_METABOLIC_PROCESS	6.22E-07	1.34E-04
MEgreen	Down	KEGG	1	KEGG_PARKINSONS_DISEASE	1.74E-08	5.23E-06
			2	KEGG_HUNTINGTONS_DISEASE	1.50E-06	2.17E-04
		GO-BP	1	GO_GOLGI_VESICLE_TRANSPORT	3.53E-09	1.33E-06
			2	GO_SMALL_MOLECULE_METABOLIC_PROCESS	5.83E-09	2.05E-06
MEroyalblue	Down	KEGG	1	KEGG_FOCAL_ADHESION	1.56E-07	3.38E-05
			2	KEGG_COMPLEMENT_AND_COAGULATION_CASCADES	2.70E-07	5.03E-05
		GO-BP	1	GO_VASCULATURE_DEVELOPMENT	2.11E-17	2.18E-14
			2	GO_BLOOD_VESSEL_MORPHOGENESIS	4.64E-17	4.65E-14
MEsalmon	Up	KEGG	1	KEGG_PROTEASOME	3.80E-06	4.36E-04
			2	KEGG_ALZHEIMERS_DISEASE	2.59E-04	0.02
		GO-BP	1	GO_MITOCHONDRION_ORGANIZATION	1.02E-11	5.64E-09
			2	GO_CELLULAR_MACROMOLECULAR_COMPLEX_ASSEMBLY	2.34E-08	7.38E-06
MEgreenyellow	Up	KEGG	1	KEGG_TAURINE_AND_HYPOTAURINE_METABOLISM	1.26E-04	0.009
			2	KEGG_BETA_ALANINE_METABOLISM	9.26E-04	0.04
		GO-BP	1	GO_CEREBRAL_CORTEX_GABAERGIC_INTERNEURON_DIFFERENTIATION	3.80E-09	1.41E-06
			2	GO_GABAERGIC_NEURON_DIFFERENTIATION	8.92E-09	3.02E-06
MEturquoise	Up	KEGG	1	KEGG_NEUROACTIVE_LIGAND_RECEPTOR_INTERACTION	6.26E-13	2.72E-10
			2	KEGG_CALCIUM_SIGNALING_PATHWAY	3.22E-09	1.14E-06
		GO-BP	1	GO_SYNAPTIC_SIGNALING	2.36E-50	6.29E-47
			2	GO_MODULATION_OF_SYNAPTIC_TRANSMISSION	6.15E-40	1.47E-36

For significant modules, results of the enrichment analysis are specified when comparing *C9orf72* expansion carriers to patients without this expansion (C9Plus vs. C9Minus). Each time, the direction of the change (Dir), process or pathway, *p*-value, and false discovery rate (FDR) are incorporated. Kyoto Encyclopedia of Genes and Genomes (KEGG) and Gene Ontology – Biological Processes (GO-BP) were used. Of note, this table has been generated after adjustment for cell-type-specific markers

The comparison between expansion carriers and controls resulted in 25 modules. Despite the fact that we adjusted for cell-type-specific markers and other potential confounders, we still observed weak correlations with those variables; for instance, due to differences in cellular composition between affected and unaffected frontal cortices (Fig. 3b). Nevertheless, the disease group displayed the strongest correlations and was significantly associated with 11 modules. An enrichment was seen for processes like GABAergic interneuron differentiation (paleturquoise), synaptic signaling (turquoise), metabolic processes (yellow), Golgi vesicle transport (green), oxidative phosphorylation (orange), protein folding (midnightblue), and cell death (steelblue; Table 6). The *C9orf72* gene was assigned to the yellow module, which we visualized (Fig. 4b); in general, expansion carriers seemed to have decreased module eigengene values for the yellow module, when comparing them to control subjects. The yellow module was enriched for various processes, including small-molecule metabolic processes (FDR = 2.10E-13), organic-acid catabolic processes (FDR = 1.39E-11), small-molecule catabolic processes (FDR = 1.15E-10), organic-acid metabolic processes (FDR = 6.24E-08), and oxidation reduction processes (FDR = 8.71E-07). The top cellular components were the mitochondrial matrix (FDR = 2.59E-10), mitochondrion (FDR = 2.18E-09), and mitochondrial part (FDR = 2.27E-09). Our gene network with top genes from significant modules highlighted genes belonging to the yellow module (Fig. 5b), such as small integral membrane protein 14 (*SMIM14*), pyrroline-5-carboxylate reductase 2 (*PYCR2*), 5′-nucleotidase domain containing 1 (*NT5DC1*), S100 calcium binding protein B (*S100B*), and dynactin subunit 6 (*DCTN6*).

**Table 6:** Enrichment Analysis (C9Plus vs. Control)

Module	Dir	Source	Order	Process/Pathway	*P*-value	FDR
MEskyblue	Up	KEGG	1	KEGG_MELANOGENESIS	4.24E-04	0.04
			2	KEGG_NOTCH_SIGNALING_PATHWAY	7.46E-04	0.06
		GO-BP	1	GO_POSITIVE_REGULATION_OF_BIOSYNTHETIC_PROCESS	5.27E-05	0.008
			2	GO_REGULATION_OF_INTRACELLULAR_STEROID_HORMONE_RECEPTOR_SIGNALING_PATHWAY	5.66E-05	0.009
MEpaleturquoise	Up	KEGG	1	KEGG_NEUROACTIVE_LIGAND_RECEPTOR_INTERACTION	1.09E-04	0.01
			2	KEGG_TAURINE_AND_HYPOTAURINE_METABOLISM	1.80E-04	0.02
		GO-BP	1	GO_CEREBRAL_CORTEX_GABAERGIC_INTERNEURON_DIFFERENTIATION	7.95E-09	3.97E-06
			2	GO_GABAERGIC_NEURON_DIFFERENTIATION	1.87E-08	8.41E-06
MEturquoise	Up	KEGG	1	KEGG_NEUROACTIVE_LIGAND_RECEPTOR_INTERACTION	4.56E-15	2.26E-12
			2	KEGG_CALCIUM_SIGNALING_PATHWAY	2.44E-06	6.06E-04
		GO-BP	1	GO_SYNAPTIC_SIGNALING	3.79E-33	1.06E-29
			2	GO_CELL_CELL_SIGNALING	3.59E-23	8.31E-20
MEyellow	Down	KEGG	1	KEGG_PEROXISOME	5.76E-07	1.71E-04
			2	KEGG_VALINE_LEUCINE_AND_ISOLEUCINE_DEGRADATION	1.60E-05	0.003
		GO-BP	1	GO_SMALL_MOLECULE_METABOLIC_PROCESS	1.44E-16	2.10E-13
			2	GO_ORGANIC_ACID_CATABOLIC_PROCESS	1.23E-14	1.39E-11
MEgreen	Down	KEGG	1	KEGG_CITRATE_CYCLE_TCA_CYCLE	6.15E-05	0.009
			2	KEGG_PYRUVATE_METABOLISM	0.002	0.11
		GO-BP	1	GO_ORGANIC_ACID_METABOLIC_PROCESS	6.24E-08	2.49E-05
			2	GO_GOLGI_VESICLE_TRANSPORT	7.76E-08	2.98E-05
MEorange	Down	KEGG	1	KEGG_PARKINSONS_DISEASE	4.93E-34	1.10E-30
			2	KEGG_OXIDATIVE_PHOSPHORYLATION	3.39E-32	5.04E-29
		GO-BP	1	GO_NUCLEOSIDE_TRIPHOSPHATE_METABOLIC_PROCESS	5.35E-29	1.46E-25
			2	GO_OXIDATIVE_PHOSPHORYLATION	1.74E-28	4.63E-25
MEtan	Down	KEGG	1	KEGG_GLYCINE_SERINE_AND_THREONINE_METABOLISM	5.51E-07	1.71E-04
			2	KEGG_FATTY_ACID_METABOLISM	6.55E-07	1.83E-04
		GO-BP	1	GO_OXIDATION_REDUCTION_PROCESS	8.53E-18	1.40E-14
			2	GO_BIOLOGICAL_ADHESION	1.39E-15	1.93E-12
MEblack‘	Up	KEGG	1	KEGG_BASAL_TRANSCRIPTION_FACTORS	0.002	0.11
			2	KEGG_SPLICEOSOME	0.02	0.48
		GO-BP	1	GO_NEGATIVE_REGULATION_OF_NITROGEN_COMPOUND_METABOLIC_PROCESS	6.04E-09	3.11E-06
			2	GO_CHROMATIN_MODIFICATION	4.90E-08	2.00E-05
MEdarkred	Down	KEGG	1	KEGG_LYSOSOME	0.004	0.18
			2	KEGG_STEROID_BIOSYNTHESIS	0.005	0.19
		GO-BP	1	GO_ENSHEATHMENT_OF_NEURONS	4.31E-05	0.007
			2	GO_NEGATIVE_REGULATION_OF_CELLULAR_COMPONENT_ORGANIZATION	5.22E-05	0.008
MEmidnightblue	Up	KEGG	1	KEGG_ANTIGEN_PROCESSING_AND_PRESENTATION	5.26E-05	0.008
			2	KEGG_SYSTEMIC_LUPUS_ERYTHEMATOSUS	0.001	0.09
		GO-BP	1	GO_PROTEIN_FOLDING	2.29E-21	4.69E-18
			2	GO_PROTEIN_REFOLDING	9.27E-12	8.16E-09
MEsteelblue	Up	KEGG	1	KEGG_CYTOKINE_CYTOKINE_RECEPTOR_INTERACTION	0.001	0.09
			2	KEGG_APOPTOSIS	0.002	0.11
		GO-BP	1	GO_INFLAMMATORY_RESPONSE	7.44E-06	0.002
			2	GO_CELL_DEATH	1.23E-05	0.002

For significant modules, results of the enrichment analysis are specified when comparing *C9orf72* expansion carriers to control subjects (C9Plus vs. Control). Each time, the direction of the change (Dir), process or pathway, p-value, and false discovery rate (FDR) are incorporated. Kyoto Encyclopedia of Genes and Genomes (KEGG) and Gene Ontology – Biological Processes (GO-BP) were used. Of note, this table has been generated after adjustment for cell-type-specific markers

Of note, without adjustment for cell-type-specific markers, the strongest relationships were no longer observed for the disease group, but for our surrogate markers (Additional file 1: Figure S1). As an example, neurons were highly correlated with the turquoise module, when comparing *C9orf72* expansion carriers to patients without this expansion (correlation: 0.82; Additional file 1: Figure S1a) or to control subjects (correlation: 0.83; Additional file 1: Figure S1b). Enrichment analysis confirmed that the turquoise module was enriched for synaptic signaling (FDR = 1.30E-53 and FDR = 2.09E-44, respectively). Similarly, microglia were strongly correlated with the grey60 module, demonstrating a correlation of 0.87 for both comparisons, while being enriched for the immune response (FDR = 8.23E-62 and FDR = 1.51E-63, respectively). The importance of our adjustment for cell-type-specific markers was further substantiated by a cluster dendrogram (Additional file 1: Figure S2); branches in this dendrogram correspond to the modules we identified. After adjustment for cellular composition (Additional file 1: Figure S2a), the turquoise module was relatively small and seemed more closely related to the disease group than to our neuronal marker. Without this adjustment, however, the turquoise module was much larger and resembled the pattern of our neuronal marker (Additional file 1: Figure S2b). Importantly, without adjustment for surrogate markers, the green module involved in vesicular transport and the yellow module that contains *C9orf72* still correlated with the disease group (Additional file 1: Figure S1 and S3), but findings were less prominent than those obtained after adjustment.

### Machine learning uncovers clinico-pathological associations

We then performed an exploratory analysis aiming at the discovery of clinico-pathological associations, when restricting our cohort to FTLD and FTLD/MND patients harboring an expanded *C9orf72* repeat (*n* = 34). Three types of models were used with residuals adjusted for cell-type-specific markers as input: linear regression models, logistic regression models, and Cox proportional hazard regression models. Our single-gene analysis did not reveal individual genes that remained significant after adjustment for multiple testing (not shown). Nonetheless, when analyzing all nominally significant genes, machine learning did point to interesting candidates, which were consistently associated with a given outcome using multiple methods and which were biologically relevant.

**Fig. 6:**
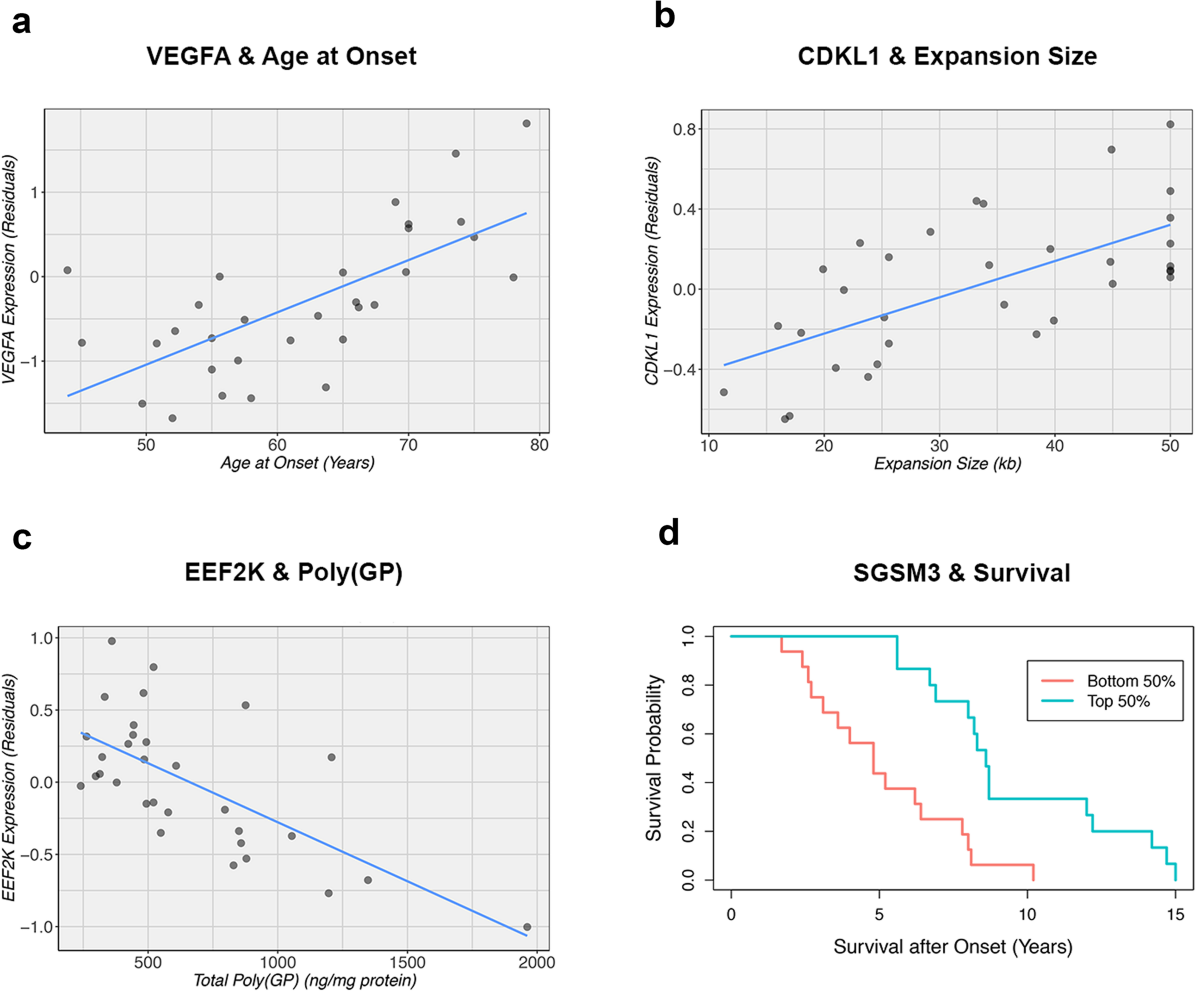
**a**-**d** Associations are displayed for patients carrying a *C9orf72* repeat expansion. **a** The first plot shows an association between *VEGFA* and age at onset. **b** An association between *CDKL1* and *C9orf72* expansion size is shown in the second plot. **c** The third plot displays an association between *EEF2K* and poly(GP) dipeptide repeat (DPR) protein levels. In these three plots, the *solid blue line* denotes the linear regression line, while each individual is represented by a *solid dark grey circle*. **d** The last plot indicates that patients with higher *SGSM3* levels demonstrate prolonged survival after onset, when comparing the bottom 50% (*solid salmon line*) to the top 50% (*solid turquoise line*). These plots have been created using residuals adjusted for differences in cellular composition

**Fig. 7:**
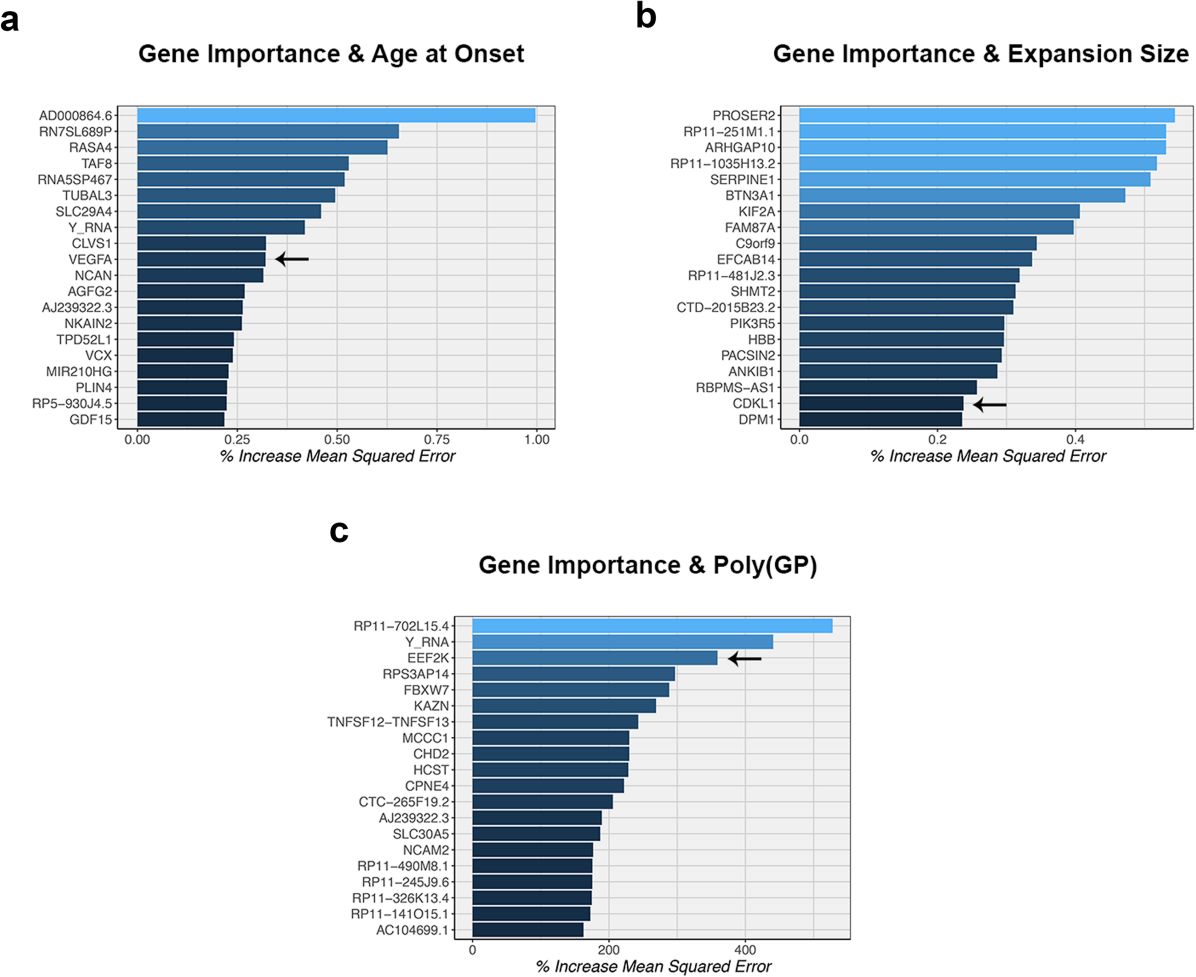
**a**-**c** The importance of genes is visualized in three plots based on a random forest analysis. For continuous variables (age at onset, *C9orf72* expansion size, and poly[GP] levels), the importance is defined as an increase in mean squared error. The *blue gradient* represents the importance of each gene, from very important (*light*) to less important (*dark*). *Arrows* point at genes of interest, namely *VEGFA*, *CDKL1*, and *EEF2K* (Table 7 and Fig. 6)

The most parsimonious models generated by LASSO regression contained up to 13 genes, depending on the variable studied (Table 7). When focusing on age at onset as response variable, for instance, only one gene was found: *VEGFA* (Fig. 6a). Importantly, this gene was the 10th gene based on our random forest analysis (Fig. 7a), and additionally, it was the 6th gene in our single-gene analysis (*P* = 9.17E-05). One of the four genes selected by LASSO regression that seemed associated with *C9orf72* expansion size was *CDKL1* (Fig. 6b). This gene was listed as the 19th gene in the random forest analysis (Fig. 7b) and the top gene in the single-gene analysis (*P* = 5.28E-05). Another interesting gene identified by LASSO regression was *EEF2K*, which appeared to be associated with the level of poly(GP) proteins (Fig. 6c). This gene was also the 3rd most important variable according to a random forest algorithm (Fig. 7c) and the 6th gene according to the single-gene analysis (*P* = 9.69E-04). Without adjustment for surrogate markers, similar trends were observed for *VEGFA* (*P* = 9.47E-04), *CDKL1* (*P* = 0.01), and *EEF2K* (*P* = 0.002; Additional file 1: Figure S4a-c).

**Table 7:** LASSO Regression

Variable	Order	LASSO			Single-Gene		
		Gene	Chr	Coef1	Coef2	SE	*P*-value
Age at Onset	1	***VEGFA***	chr6	0.14	7.36	1.61	9.17E-05
Age at Death	1	*AUTS2*	chr7	0.68	23.86	4.64	1.41E-05
	2	*TAF8*	chr6	7.00	28.34	6.58	1.55E-04
	3	*NAGS*	chr17	0.16	10.87	2.58	2.02E-04
	4	*RP1-130H16.18*	chr22	2.88	15.96	3.82	2.23E-04
	5	*DOCK10*	chr2	-1.31	-13.55	3.26	2.38E-04
	6	*CLEC4E*	chr12	0.56	4.53	1.09	2.43E-04
	7	*APOBR*	chr16	0.88	6.59	1.60	2.71E-04
	8	*LYST*	chr1	-5.12	-29.58	7.43	3.86E-04
	9	*SLC39A11*	chr17	0.25	13.82	3.48	3.96E-04
	10	*RP11-3G21.1*	chr8	0.29	6.95	1.78	4.67E-04
	11	*TIMM13*	chr19	-0.38	-22.98	6.48	0.001
	12	*VCX*	chrX	3.39	12.55	3.56	0.001
	13	*SELPLG*	chr12	0.05	6.55	2.08	0.004
Expansion Size	1	***CDKL1***	chr14	3.06	21.39	4.52	5.28E-05
	2	*FAM87A*	chr8	-0.50	-9.21	2.24	2.94E-04
	3	*RP11-481 J2.3*	chr16	0.64	7.52	2.14	0.001
	4	*EFCAB14*	chr1	-2.38	-34.03	9.98	0.002
Sense RNA Foci	1	*CATIP-AS1*	chr2	-0.002	-0.10	0.02	6.34E-05
Antisense RNA Foci	1	*CCDC127*	chr5	0.003	0.16	0.04	0.001
Poly(GP)	1	***EEF2K***	chr16	-10.11	- 495.81	133.94	9.69E-04
Methylation	1	*AC006946.16*	chr22	-0.03	-8.14	2.05	4.32E-04
	2	*RP11-555 M1.3*	chr3	0.07	4.18	1.33	0.004
	3	*A1BG*	chr19	-0.009	-8.69	2.78	0.004
Disease Subgroup	1	*TMPPE*	chr3	0.02	3.03	1.10	0.006
	2	*EDEM2*	chr20	0.93	8.44	3.13	0.007
	3	*CD37*	chr19	-0.39	-6.42	2.41	0.008
	4	*MZT2B*	chr2	-1.15	-22.86	8.75	0.009
	5	*PLK5*	chr19	-0.02	-2.52	1.00	0.01
	6	*NOTUM*	chr17	-0.32	-4.87	2.30	0.03
Variable	Order	Gene	Chr	Coef1	HR	CI	*P*-value
Survival after Onset	1	***SGSM3***	chr22	-0.02	0.10	0.04–0.28	1.31E-05
	2	*EFNA2*	chr19	-0.03	0.19	0.07–0.51	0.001

Genes selected using Least Absolute Shrinkage and Selection Operator (LASSO) regression are shown for variables of interest. The chromosome (Chr) and LASSO coefficient (Coef1) are included. Additionally, the results of the single-gene analysis are displayed for genes identified through LASSO regression, including the coefficient (Coef2), standard error (SE), hazard ratio (HR), 95% confidence interval (CI), and/or *p*-value. Genes denoted by a bold font are displayed in Fig. 6. Of note, this table has been generated after adjustment for cell-type-specific markers

In the survival after onset model, LASSO regression identified two genes, one of which was a gene called *SGSM3* that was the top hit of our single-gene analysis (*P* = 1.31E-05; Table 7). In patients belonging to the bottom 50% of *SGSM3* expression levels, the median survival after onset was 4.8 years (IQR: 3.0–6.8) versus 8.6 years in the top 50% (IQR: 7.5–12.1; Fig. 6d). This difference resulted in an HR of 0.10 (95% CI: 0.04–0.28). We were able to confirm these findings when analyzing expression levels based on rank, listing *SGSM3* as the 3rd gene (*P* = 6.03E-04). Likewise, when treating expression levels as a continuous variable, *SGSM3* was the 13th gene on the list (*P* = 0.001). Although much less profound, this trend with survival after onset was also observed without adjustment for cell-type-specific markers (*P* = 0.02; Additional file 1: Figure S4d). Together, our findings suggest that lower levels of *SGSM3* might be associated with shortened survival after onset in *C9orf72* expansion carriers. Notably, of our four genes of interest, *SGSM3* was the only gene that was significantly differentially expressed between disease groups (FDR = 0.03), demonstrating elevated levels in patients carrying an expanded *C9orf72* repeat (Additional file 1: Figure S5).

We then used TaqMan expression assays for the four top candidate genes to validate the expression results from our RNA sequencing experiment in *C9orf72* expansion carriers. When using residuals unadjusted for cellular composition, a significant correlation between our expression assays and RNA sequencing data was found for *VEGFA* (*P* = 4.17E-05, correlation: 0.68), *CDKL1* (*P* = 0.003, correlation: 0.55), *EEF2K* (*P* = 0.03, correlation: 0.40), and *SGSM3* (*P* = 0.03, correlation: 0.40; Additional file 1: Figure S6b, d, f, h). Similar correlations were obtained when using residuals adjusted for our five surrogate markers (Additional file 1: Figure S6a, c, e, g).

## Discussion

In this study, we characterized the expression pattern of *C9orf72*-related diseases in an affected brain region: the frontal cortex. We examined FTLD and FTLD/MND patients with or without a *C9orf72* repeat expansion as well as control subjects (*n* = 102). Differential expression analysis identified *C9orf72* as the top gene; it was approximately 50% reduced in *C9orf72* expansion carriers. Importantly, differentially expressed genes were enriched for endocytosis (FDR = 0.02). Without adjustment for cell-type-specific markers, our co-expression analysis revealed modules influenced by neuronal loss (turquoise) and inflammation (grey60). Usage of surrogate markers resulted in the discovery of additional modules that correlated with the disease group, including modules enriched for protein folding, RNA processing, metabolic processes, and vesicle-mediated transport. The *C9orf72* gene itself was assigned to a module involved in metabolism (yellow) and clustered with genes belonging to a module that plays a role in vesicular transport (green). To identify potential disease modifiers, we then focused on the subset of individuals with an expanded repeat in *C9orf72* (*n* = 34). We used various analytical approaches, including LASSO regression and random forest, which pointed to promising candidates. In addition to *VEGFA*, for instance, we detected *CDKL1*, *EEF2K*, and *SGSM3*. Taken together, our RNA sequencing study uncovered that vital processes, such as vesicle transport, are affected by the presence of a repeat expansion in *C9orf72*. Furthermore, the modifiers identified in this study may represent biomarkers and/or therapeutic targets, which are in great demand.

Although the C9orf72 protein has been studied extensively since the discovery of a repeat expansion in the *C9orf72* gene [14, 50], little is known about its function. It has been suggested that C9orf72 is a member of a superfamily called differentially expressed in normal and neoplasia (DENN) [36, 65], which contains GDP/GTP exchange factors (GEFs) that activate regulators of membrane trafficking known as Rab-GTPases. The C9orf72 protein has already been shown to co-localize with Rab-GTPases involved in endosomal transport [18]. Additionally, C9orf72 was found to form a complex with another DENN protein (SMCR8), serving as a GEF for specific Rab-GTPases [2, 53, 62, 64]. Furthermore, the C9orf72 protein appears to play a role in lysosomal biogenesis in addition to vesicle trafficking [56]. The presence of the *C9orf72* repeat expansion seems to cause defects in vesicle trafficking and dysfunctional trans-Golgi network phenotypes, which can be reversed by overexpression of *C9orf72* or antisense oligonucleotides targeting the expanded repeat [3]. Interestingly, modulation of vesicle trafficking may even rescue neurodegeneration in induced motor neurons from *C9orf72* expansion carriers [56].

Our study, in which we compared the expression pattern of *C9orf72* expansion carriers to (disease) controls, uncovered *C9orf72* as the top hit of our differential expression analysis. This aligns with one of our previous studies where we detected reduced levels of *C9orf72* transcripts in expansion carriers and where we observed clinico-pathological associations with specific transcript variants [59]. It was reassuring to see that differentially expressed genes were enriched for endocytosis, especially given the potential role of the C9orf72 protein in vesicular transport. These findings were further substantiated by the fact that our co-expression analysis revealed a module that was enriched for Golgi vesicle transport as well as endoplasmic reticulum to Golgi vesicle-mediated transport, vacuolar transport, vesicle-mediated transport, and lysosomes. Our RNA sequencing study, therefore, provides additional evidence that the presence of a *C9orf72* repeat expansion might disrupt vesicle trafficking, a crucial process. Interestingly, we also discovered a promising modifier of survival after onset that is involved in vesicle transport: *SGSM3*. Our findings indicate that low expression levels of *SGSM3* could be detrimental in *C9orf72* expansion carriers, while high levels might have protective effects. The SGSM3 protein interacts with Ras-related protein Rab-8A [63], a small Rab-GTPase that is also regulated by the C9orf72-SMCR8 complex [53]. Consequently, one could postulate that higher levels of *SGSM3* might counteract some of the harmful effects associated with an expanded repeat in *C9orf72*. In fact, a recent yeast screen demonstrated that *msb3*, the yeast ortholog of *SGSM3*, modifies the toxicity of one of the DPR proteins: poly(GR) [9]; other potential mechanisms seem worthy of exploration.

Another interesting candidate we identified, *VEGFA*, appeared to be associated with the age at which disease symptoms occur. Our findings suggest that higher expression levels of this gene are associated with a delayed age at onset (*P* = 9.17E-05, coefficient: 7.36). While age at onset and age at death are strongly correlated, one could speculate that *VEGFA* levels might simply increase as an individual ages. Our single-gene analysis, however, revealed a stronger association with age at onset than with age at death (*P* = 0.003, coefficient: 5.81). The VEGFA protein belongs to the vascular endothelial growth factor (VEGF) family and is thought to have neurotrophic effects [28, 29]. Remarkably, reduced expression of *Vegfa* has been shown to cause an ALS-like phenotype in mice [45]. At the same time, treatment with Vegfa might protect motor neurons against ischemic death [32]. Additionally, genetic variants in *VEGFA* may render individuals more vulnerable to the development of ALS [31, 32]. Notably, neither an association with survival after onset (*P* = 0.26) nor a significant difference between disease subgroups (FTLD versus FTLD/MND; *P* = 0.75) was observed in our *C9orf72* expansion carriers, but the association we detected with age at onset is in favor of a protective role for VEGFA.

In addition to *SGSM3* and *VEGFA*, we also found associations with *CDKL1* and *EEF2K*. *CDKL1* was associated with the size of *C9orf72* expansions: higher levels were observed in individuals with longer expansions. This gene is a member of the cyclin-dependent kinase family and appears to control the length of neuronal cilia [8]. At the moment, how *CDKL1* possibly affects *C9orf72* expansion size remains elusive. Expression levels of *EEF2K* were associated with the amount of poly(GP); an increase in *EEF2K* was seen in expansion carriers when poly(GP) levels decreased. It is a regulator of protein synthesis and synaptic plasticity that has already been studied in Alzheimer’s disease and Parkinson’s disease, where it may affect the toxicity of amyloid-β and α-synuclein [25–27]. Given the fact that it functions in protein synthesis and has previously been implicated in other neurodegenerative diseases, *EEF2K* is an interesting candidate. Of note, for simplicity, we focused on four disease modifiers in this manuscript; however, our study also hints at the involvement of other genes (e.g., Table 7), which might be worth pursuing.

It should be noted that, although we performed RNA sequencing on a precious collection of well-characterized individuals for whom autopsy tissue was available, the actual number of samples included in our study is limited. This mainly affects the clinico-pathological association analyses performed in the subset of individuals carrying an expanded *C9orf72* repeat; these analyses, therefore, should be considered exploratory in nature. Additionally, we would like to stress that patients included in this study were generally younger than control subjects. Despite the fact that we adjusted our models for age at death, we realize that this age difference may have influenced our findings. Another limitation that should be mentioned is that we performed RNA sequencing on bulk tissue from the frontal cortex instead of on single nuclei. Because expression levels are cell-type dependent, we included five genes in our models as surrogate markers [1, 12, 23]. Evidently, this approach is not perfect, but it enabled us to (partially) account for various degrees of neuronal loss, inflammation, and gliosis seen in patients with FTLD and/or MND. When taking the cost of single nuclei RNA sequencing into consideration, our bulk tissue analysis with adjustment for cellular composition seems to provide a cost-effective alternative that can yield significant results. Future studies could further investigate expression levels of interesting candidates in specific cell types to elucidate which cells are most relevant for a given gene and appear to drive the detected associations (e.g., using purified cell populations), and additionally, they could clarify whether changes on the protein level mirror changes on the RNA level.

## Conclusions

To conclude, in this study, we have used a combination of conventional analyses and machine learning to capture the RNA signature of *C9orf72*-linked diseases. Our powerful approach highlights the disruptive effects of a repeat expansion in *C9orf72*, particularly on vesicular transport. Furthermore, we have discovered promising candidate modifiers that were consistently associated with relevant disease features and that may serve as urgently needed biomarkers and/or point to new treatment strategies.

## Additional file

Additional file 1: **Figure S1 a** Module-trait relationships are presented for patients with an expanded *C9orf72* repeat and patients without this repeat (C9Plus vs. C9Minus). **b** For patients with an expansion and control subjects (C9Plus vs. Control), module-trait relationships are plotted. These plots are generated with weighted gene co-expression network analysis (WGCNA) to find groups of genes that go up (*red*) or down (*blue*) together. A unique color has been assigned to each of these groups, also called a module. Correlations and p-values are shown for variables of interest, including disease group (C9Plus, C9Minus, and/or Control; *arrow*), neurons, microglia, astrocytes, oligodendrocytes, endothelial cells, RNA integrity number (RIN), age at death, sex, and plate. The strongest correlations (*brightest colors*) are observed for cell types. Notably, both module-trait relationship plots are based on residuals obtained without adjustment for cell-type-specific markers. **Figure S2 a** With adjustment for cell-type-specific markers, a cluster dendrogram is shown for *C9orf72* expansion carriers and control subjects. **b** For the same comparison, a cluster dendrogram is displayed without adjustment for cell-type-specific markers. The branches in these dendrograms correspond to specific modules. A unique color has been assigned to each of these modules. Additionally, variables of interest are included, such as the disease group, neurons, microglia, astrocytes, oligodendrocytes, endothelial cells, RNA integrity number (RIN), age at death, sex, and plate. High levels are shown in *red* and low levels in *blue*. After adjustment, no striking differences are observed based on cell type; without adjustment, however, modules appear to be associated with certain cell types (e.g., turquoise and neurons). **Figure S3 a** For patients harboring a *C9orf72* repeat expansion and those without this expansion (C9Plus vs. C9Minus; module membership > 0.6 and significance < 1.0E-05), a gene network is displayed. **b** A gene network is visualized when examining expansion carriers and controls (C9Plus vs. Control; module membership > 0.6 and significance < 1.0E-05). In these network plots, the connectivity of each gene is represented by the *size* of its node, the module to which it has been assigned by its *color*, and the strength of the correlation by the *thickness* of its edges; the *C9orf72* gene is denoted by an *arrow*. Of note, the plots in this figure have been generated without adjustment for cell-type-specific markers. **Figure S4 a-d** Trends are displayed for patients carrying a *C9orf72* repeat expansion. **a** The first plot shows *VEGFA* and age at onset. **b** *CDKL1* and *C9orf72* expansion size are shown in the second plot. **c** The third plot displays *EEF2K* and poly(GP) levels. In these three plots, the *solid blue line* denotes the linear regression line, while each individual is represented by a *solid dark grey circle*. **d** The last plot shows *SGSM3* levels and survival after onset, when comparing the bottom 50% (*solid salmon line*) to the top 50% (*solid turquoise line*). These plots have been created using residuals unadjusted for differences in cellular composition. **Figure S5 a-h** The expression levels of *VEGFA*, *CDKL1*, *EEF2K*, and *SGSM3* are shown for all disease groups: patients with a *C9orf72* repeat expansion (C9Plus), patients without this expansion (C9Minus), and control subjects (Control), both with and without adjustment for cell-type-specific markers. For each box plot, the median is represented by a *solid black line*, and each box spans the interquartile range (IQR; 25th percentile to 75th percentile). **Figure S6 a-h** This figure displays the correlation between our expression assays (relative expression) and RNA sequencing data (residuals). **a-b** The first two plots show correlations for *VEGFA*, either with or without adjustment for cell-type-specific markers. **c-d** The next two plots visualize correlations for *CDKL1*, both with and without adjustment for cellular composition. **e-f** *EEF2K* is displayed on the next plots, again with and without adjustment for surrogate markers. **g-h** The last two plots show correlations for *SGSM3* with and without adjustment for cellular composition. For each of these plots, the *solid blue line* denotes the linear regression line, while each individual is represented by a *solid dark grey circle*. (PDF 2894 kb)

## Data Availability

Upon reasonable request, data and/or scripts used for this study will be shared by the corresponding authors.

## Abbreviations

ALS: Amyotrophic lateral sclerosis
bicor: Biweight midcorrelation
*C9orf72*: C9orf72-SMCR8 complex subunit
*CD34*: CD34 molecule
*CD68*: CD68 molecule
*CDKL1*: Cyclin dependent kinase like 1
*CHMP2B*: Charged multivesicular body protein 2B
CI: Confidence interval
CQN: Conditional quantile normalization
*DCTN6*: Dynactin subunit 6
DENN: Differentially expressed in normal and neoplasia
DPR: Dipeptide repeat
*EEF2K*: Eukaryotic elongation factor 2 kinase
*ENO2*: Enolase 2
FDR: False discovery rate
FTD: Frontotemporal dementia
FTLD: Frontotemporal lobar degeneration
GEF: GDP/GTP exchange factor
*GFAP*: Glial fibrillary acidic protein
HR: Hazard ratio
iPSC: Induced pluripotent stem cell
IQR: Interquartile range
LASSO: Least Absolute Shrinkage and Selection Operator
LINE: Long interspersed nuclear element
MND: Motor neuron disease
MSigDB: Molecular signatures database
*NT5DC1*: 5′-nucleotidase domain containing 1
*OLIG2*: Oligodendrocyte transcription factor 2
*PYCR2*: Pyrroline-5-carboxylate reductase 2
RIN: RNA integrity number
RPKM: Reads per kb per million
*S100B*: S100 calcium binding protein B
*SGSM3*: Small G protein signaling modulator 3
*SMIM14*: Small integral membrane protein 14
*SOD1*: Superoxide dismutase 1
SOV: Source of variation
STAR: Spliced Transcripts Alignment to a Reference
TDP-43: TAR DNA-binding protein 43
*VEGFA*: Vascular endothelial growth factor A
WGCNA: Weighted gene co-expression network analysis
