# Supplementary material for: Extensive transcriptomic study emphasizes importance of vesicular transport in C9orf72 expansion carriers
Source: Acta Neuropathol Commun. 2019 Oct 8;7:150. doi: 10.1186/s40478-019-0797-0 (PMC6781370; doi:10.1186/s40478-019-0797-0)
Supplement: Supplementary file 1 — Figure S1 a Module-trait relationships are presented for patients with an expanded C9orf72 repeat and patients without this repeat (C9Plus vs. C9Minus). b For patients with an expansion and control subjects (C9Plus vs. Control), module-trait relationships are plotted. These plots are generated with weighted gene co-expression network analysis (WGCNA) to find groups of genes that go up (red) or down (blue) together. A unique color has been assigned to each of these groups, also called a module. Correlations and p-values are shown for variables of interest, including disease group (C9Plus, C9Minus, and/or Control; arrow), neurons, microglia, astrocytes, oligodendrocytes, endothelial cells, RNA integrity number (RIN), age at death, sex, and plate. The strongest correlations (brightest colors) are observed for cell types. Notably, both module-trait relationship plots are based on residuals obtained without adjustment for cell-type-specific markers. Figure S2 a With adjustment for cell-type-specific markers, a cluster dendrogram is shown for C9orf72 expansion carriers and control subjects. b For the same comparison, a cluster dendrogram is displayed without adjustment for cell-type-specific markers. The branches in these dendrograms correspond to specific modules. A unique color has been assigned to each of these modules. Additionally, variables of interest are included, such as the disease group, neurons, microglia, astrocytes, oligodendrocytes, endothelial cells, RNA integrity number (RIN), age at death, sex, and plate. High levels are shown in red and low levels in blue. After adjustment, no striking differences are observed based on cell type; without adjustment, however, modules appear to be associated with certain cell types (e.g., turquoise and neurons). Figure S3 a For patients harboring a C9orf72 repeat expansion and those without this expansion (C9Plus vs. C9Minus; module membership > 0.6 and significance < 1.0E-05), a gene network is displayed. b A gene networ [file 40478_2019_797_MOESM1_ESM.pdf]

Electronic Supplementary Material

Extensive transcriptomic study emphasizes importance of vesicular transport in *C9orf72* expansion carriers

Authors: Dennis W. Dickson, Matthew C. Baker, Jazmyne L. Jackson, Mariely DeJesus-Hernandez, NiCole A. Finch, Shulan Tian, Michael G. Heckman, Cyril Pottier, Tania F. Gendron, Melissa E. Murray, Yingxue Ren, Joseph S. Reddy, Neill R. Graff-Radford, Bradley F. Boeve, Ronald C. Petersen, David S. Knopman, Keith A. Josephs, Leonard Petrucelli, Björn Oskarsson, John W. Sheppard, Yan W. Asmann, Rosa Rademakers, and Marka van Blitterswijk

Corresponding authors: Marka van Blitterswijk, MD, PhD, Department of Neuroscience, Mayo Clinic, 4500 San Pablo Road, Jacksonville, FL 32224, USA. Telephone number: +1 904 953 2226. Fax number: +1 904 953 7370. Email: VanBlitterswijk.Marka@mayo.edu; Rosa Rademakers, PhD, Department of Neuroscience, Mayo Clinic, 4500 San Pablo Road, Jacksonville, FL 32224, USA. Telephone number: +1 904 953 6279. Fax number: +1 904 953 7370. Email: Rademakers.Rosa@mayo.edu.

Contents: Additional file 1: Figure S1–S6

Figure Legends

Figure S1 a Module-trait relationships are presented for patients with an expanded *C9orf72* repeat and patients without this repeat (C9Plus vs. C9Minus). **b** For patients with an expansion and control subjects (C9Plus vs. Control), module-trait relationships are plotted. These plots are generated with weighted gene co-expression network analysis (WGCNA) to find groups of genes that go up (*red*) or down (*blue*) together. A unique color has been assigned to each of these groups, also called a module. Correlations and p-values are shown for variables of interest, including disease group (C9Plus, C9Minus, and/or Control; *arrow*), neurons, microglia, astrocytes, oligodendrocytes, endothelial cells, RNA integrity number (RIN), age at death, sex, and plate. The strongest correlations (*brightest colors*) are observed for cell types. Notably, both module-trait relationship plots are based on residuals obtained without adjustment for cell-type-specific markers.

Figure S 2. a With adjustment for cell-type-specific markers, a cluster dendrogram is shown for *C9orf72* expansion carriers and control subjects. **b** For the same comparison, a cluster dendrogram is displayed without adjustment for cell-type-specific markers. The branches in these dendrograms correspond to specific modules. A unique color has been assigned to each of these modules. Additionally, variables of interest are included, such as the disease group, neurons, microglia, astrocytes, oligodendrocytes, endothelial cells, RNA integrity number (RIN), age at death, sex, and plate. High levels are shown in *red* and low levels in *blue*. After adjustment, no striking differences are observed based on cell type; without adjustment, however, modules appear to be associated with certain cell types (e.g., turquoise and neurons).

Figure S3. a For patients harboring a *C9orf72* repeat expansion and those without this expansion (C9Plus vs. C9Minus; module membership > 0.6 and significance < 1.0E-05), a gene network is displayed. **b** A gene network is visualized when examining expansion carriers and controls (C9Plus vs. Control; module membership > 0.6 and significance < 1.0E-05). In these network plots, the connectivity of each gene is represented by the *size* of its node, the module to which it has been assigned by its *color*, and the strength of the correlation by the *thickness* of its edges; the *C9orf72* gene is denoted by an *arrow*. Of note, the plots in this figure have been generated without adjustment for cell-type-specific markers.

Figure S4 a-d Trends are displayed for patients carrying a *C9orf72* repeat expansion. **a** The first plot shows *VEGFA* and age at onset. **b** *CDKL1* and *C9orf72* expansion size are shown in the second plot. **c** The third plot displays *EEF2K*

and poly(GP) levels. In these three plots, the *solid blue line* denotes the linear regression line, while each individual is represented by a *solid dark grey circle*. **d** The last plot shows *SGSM3* levels and survival after onset, when comparing the bottom 50% (*solid salmon line*) to the top 50% (*solid turquoise line*). These plots have been created using residuals unadjusted for differences in cellular composition.

Figure S5 a-h The expression levels of *VEGFA*, *CDKL1*, *EEF2K*, and *SGSM3* are shown for all disease groups: patients with a *C9orf72* repeat expansion (C9Plus), patients without this expansion (C9Minus), and control subjects (Control), both with and without adjustment for cell-type-specific markers. For each box plot, the median is represented by a *solid black line*, and each box spans the interquartile range (IQR; 25th percentile to 75th percentile).

Figure S6 a-h This figure displays the correlation between our expression assays (relative expression) and RNA sequencing data (residuals). **a-b** The first two plots show correlations for *VEGFA*, either with or without adjustment for cell-type-specific markers. **c-d** The next two plots visualize correlations for *CDKL1*, both with and without adjustment for cellular composition. **e-f** *EEF2K* is displayed on the next plots, again with and without adjustment for surrogate markers. **g-h** The last two plots show correlations for *SGSM3* with and without adjustment for cellular composition. For each of these plots, the *solid blue line* denotes the linear regression line, while each individual is represented by a *solid dark grey circle*.

Figure S1

a

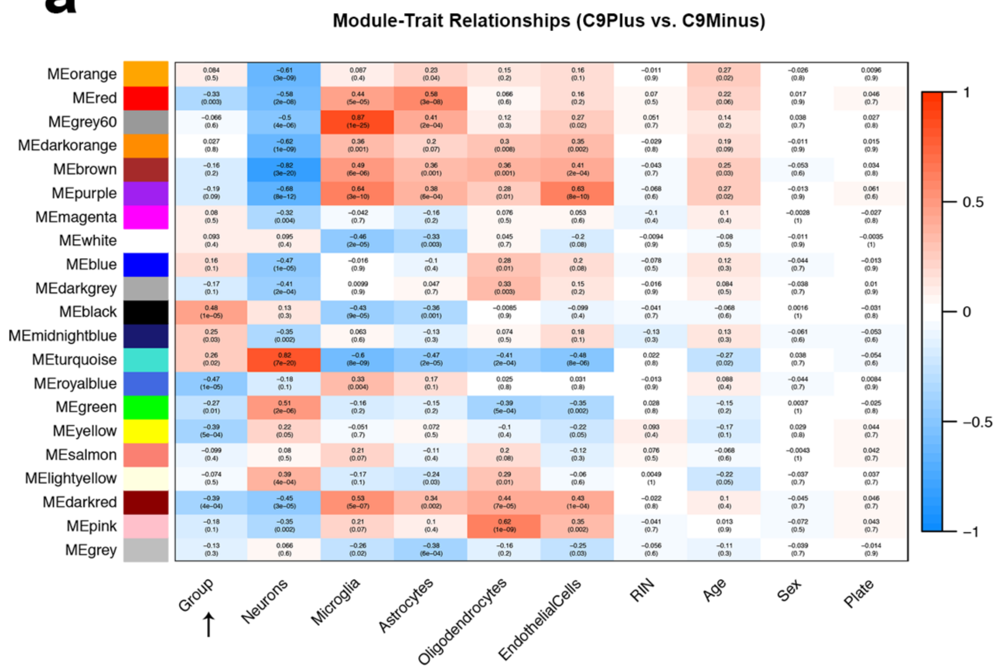
Module-Trait Relationships (C9Plus vs. C9Minus)
MEorange
MEred
MEgrey60
MEdarkorange
MEbrown
MEpurple
MEmagenta
MEwhite
MEblue
MEdarkgrey
MEblack
MEmidnightblue
MEturquoise
MEroyalblue
MEgreen
MEyellow
MEsalmon
MElightyellow
MEdarkred
MEpink
MEgrey
Group
Neurons
Microglia
Astrocytes
Oligodendrocytes
EndothelialCells
RIN
Age
Sex
Plate
1
0.5
0
−0.5
−1

b

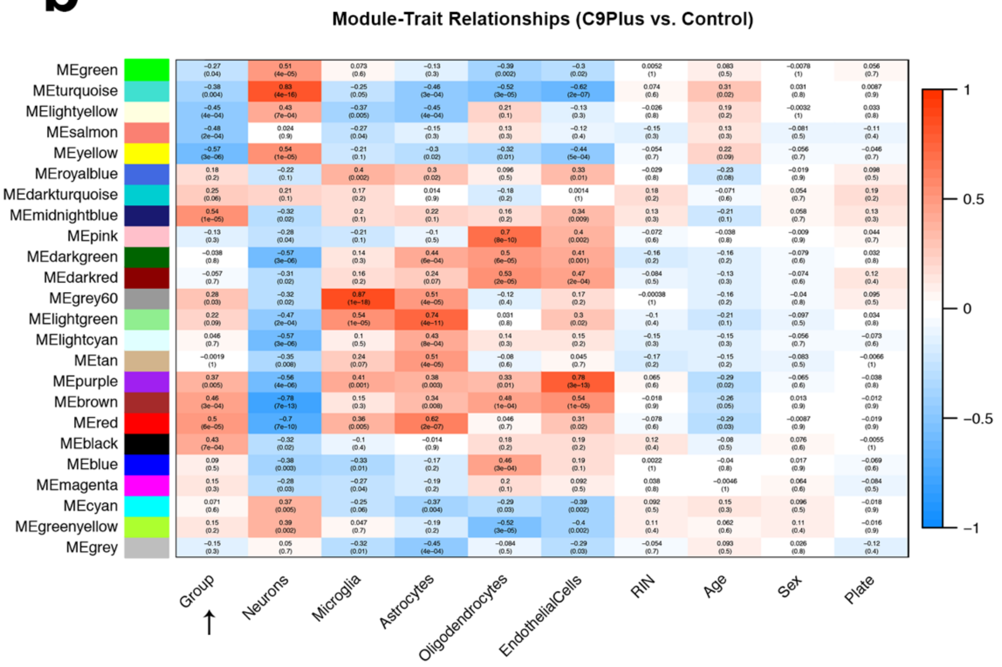
Module-Trait Relationships (C9Plus vs. Control)
MEgreen
MEturquoise
MElightyellow
MEsalmon
MEyellow
MEroyalblue
MEdarkturquoise
MEmidnightblue
MEpink
MEdarkgreen
MEdarkred
MEgrey60
MElightgreen
MElightcyan
MEtan
MEpurple
MEbrown
MEred
MEblack
MEblue
MEmagenta
MEcyan
MEgreenyellow
MEgrey
Group
Neurons
Microglia
Astrocytes
Oligodendrocytes
EndothelialCells
RIN
Age
Sex
Plate
1
0.5
0
−0.5
−1

Figure S2

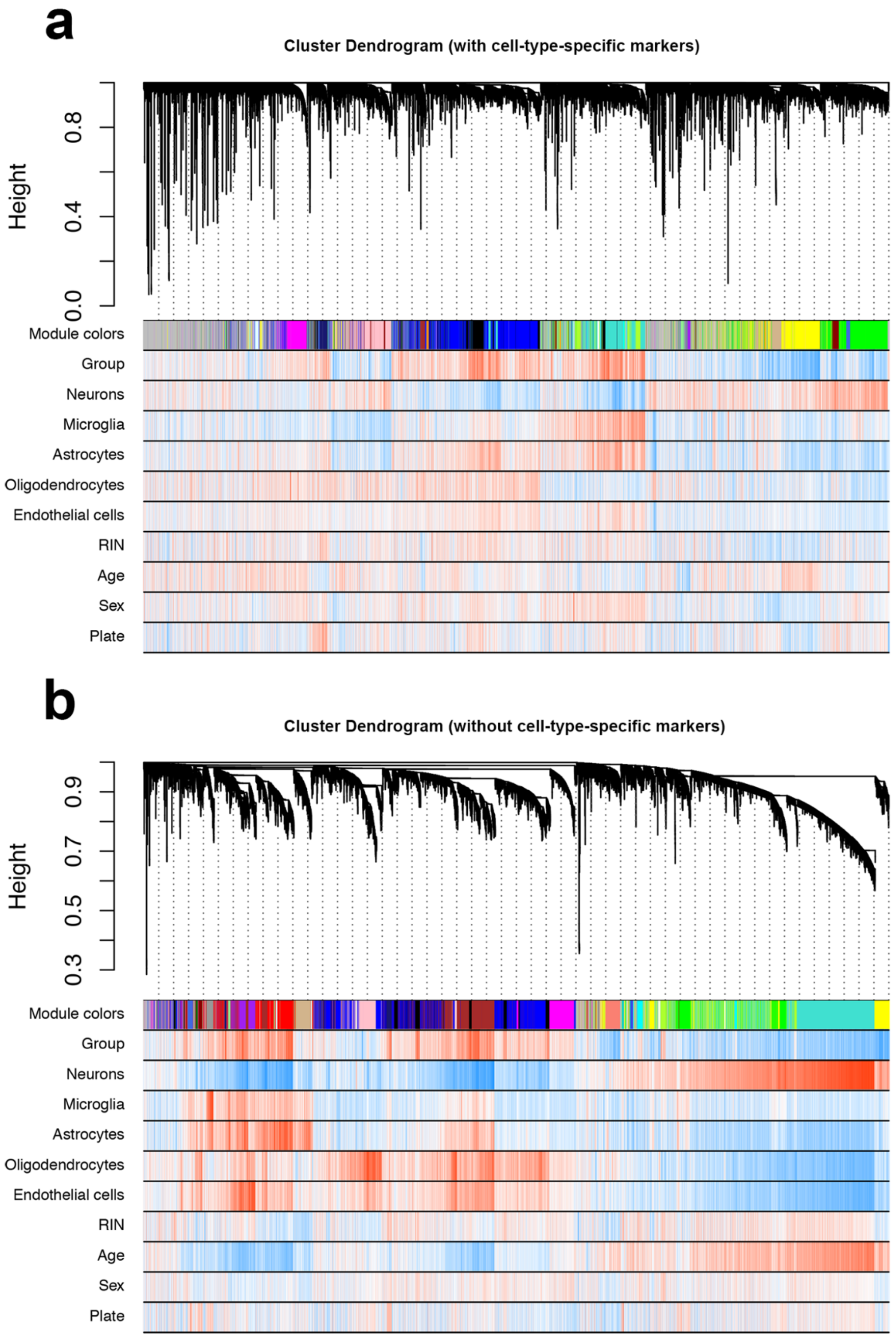
a
Cluster Dendrogram (with cell-type-specific markers)
Height
0.0
0.4
0.8
Module colors
Group
Neurons
Microglia
Astrocytes
Oligodendrocytes
Endothelial cells
RIN
Age
Sex
Plate
b
Cluster Dendrogram (without cell-type-specific markers)
Height
0.3
0.5
0.7
0.9
Module colors
Group
Neurons
Microglia
Astrocytes
Oligodendrocytes
Endothelial cells
RIN
Age
Sex
Plate

Figure S3

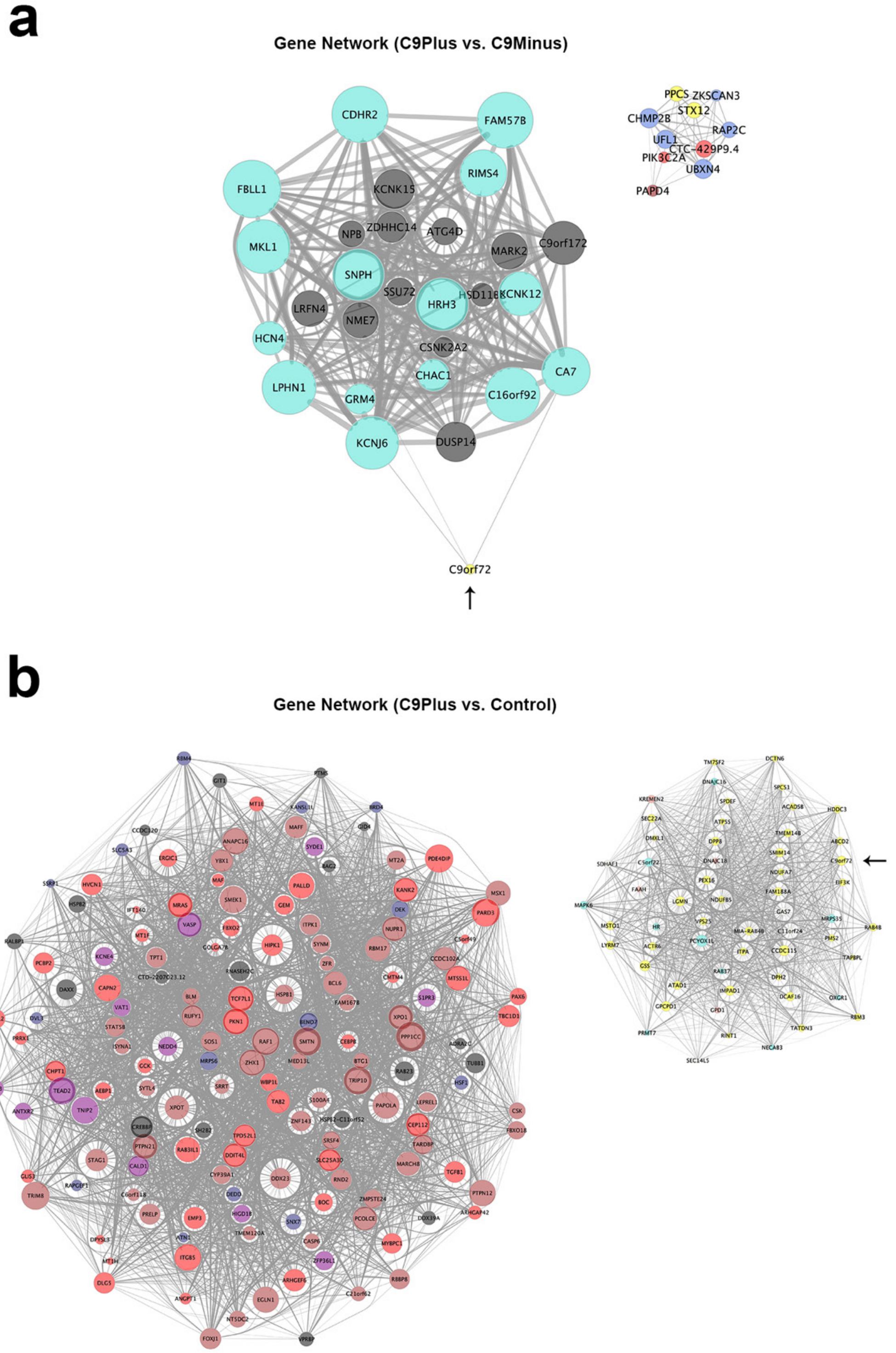
a
Gene Network (C9Plus vs. C9Minus)
CDHR2
FAM57B
FBLL1
RIMS4
KCNK15
NPB
ZDHHC14
ATG4D
C9orf172
MARK2
MKL1
SNPH
SSU72
HRH3
KCNK12
LRFN4
NME7
HCN4
CSNK2A2
CA7
CHAC1
LPHN1
GRM4
C16orf92
KCNJ6
DUSP14
C9orf72
PPCS
ZKSCAN3
STX12
CHMP2B
RAP2C
UFL1
CTC-429P9.4
PIK3C2A
UBXN4
PAPD4
b
Gene Network (C9Plus vs. Control)

Figure S4

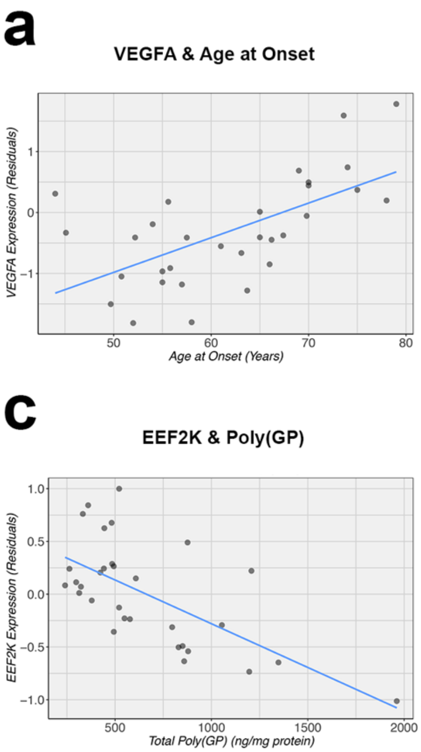
a
VEGFA & Age at Onset
VEGFA Expression (Residuals)
Age at Onset (Years)
b
CDKL1 & Expansion Size
CDKL1 Expression (Residuals)
Expansion Size (kb)
c
EEF2K & Poly(GP)
EEF2K Expression (Residuals)
Total Poly(GP) (ng/mg protein)
d
SGSM3 & Survival
Survival Probability
Survival after Onset (Years)
Bottom 50%
Top 50%

Figure S5

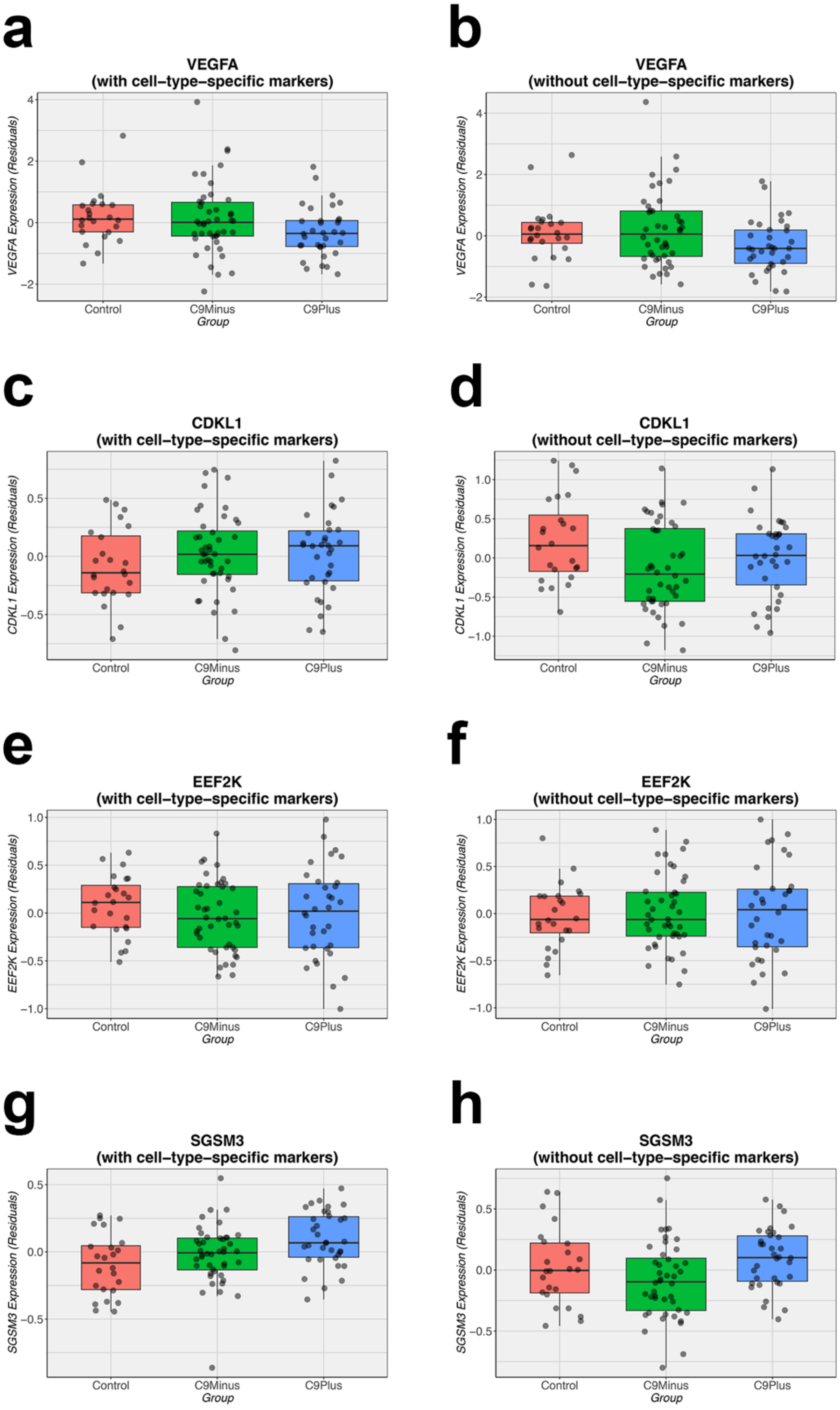
a
VEGFA
(with cell−type−specific markers)
VEGFA Expression (Residuals)
Control
C9Minus
C9Plus
Group
b
VEGFA
(without cell−type−specific markers)
VEGFA Expression (Residuals)
c
CDKL1
(with cell−type−specific markers)
CDKL1 Expression (Residuals)
d
CDKL1
(without cell−type−specific markers)
e
EEF2K
(with cell−type−specific markers)
EEF2K Expression (Residuals)
f
EEF2K
(without cell−type−specific markers)
g
SGSM3
(with cell−type−specific markers)
SGSM3 Expression (Residuals)
h
SGSM3
(without cell−type−specific markers)

Figure S6

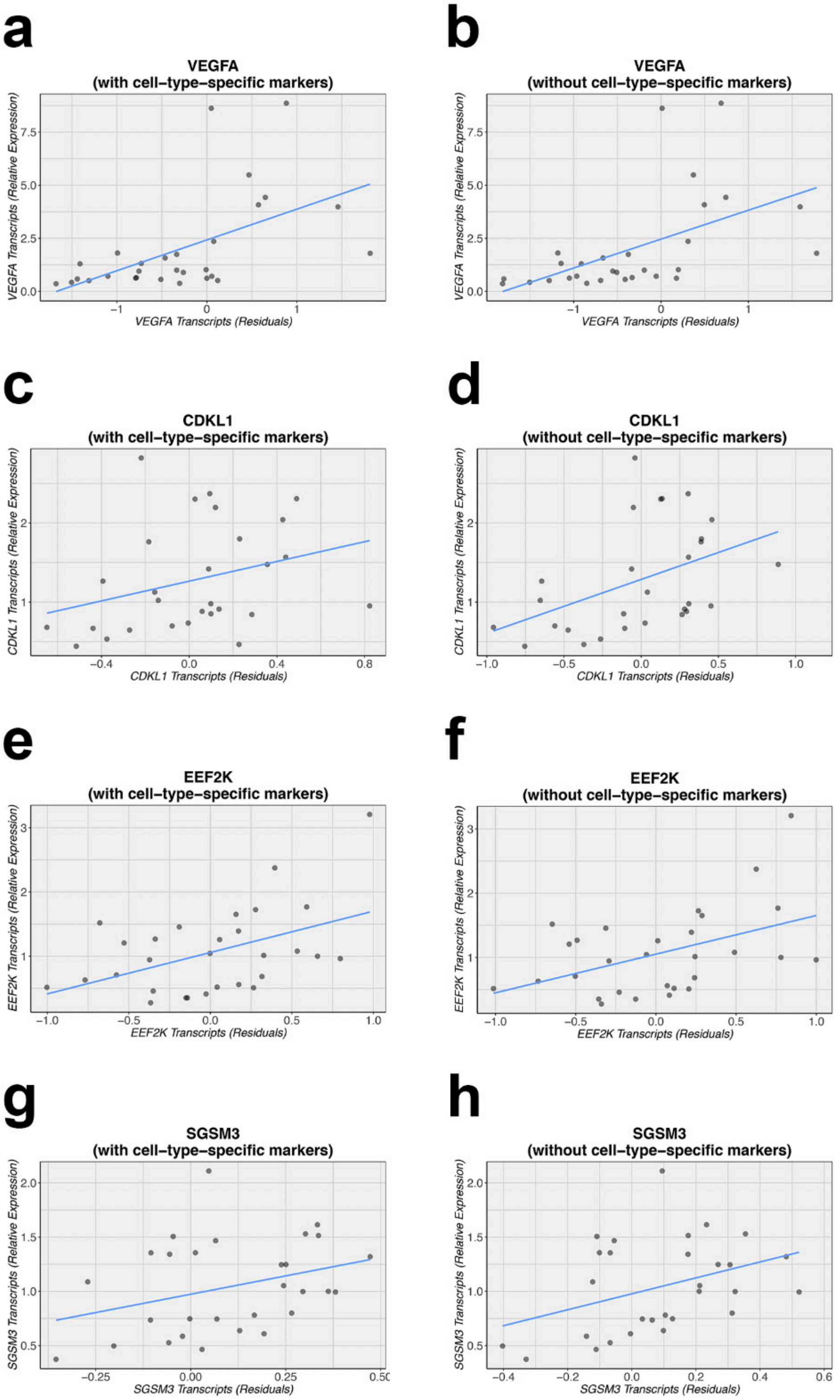
a
VEGFA
(with cell-type-specific markers)
VEGFA Transcripts (Relative Expression)
VEGFA Transcripts (Residuals)
b
VEGFA
(without cell-type-specific markers)
VEGFA Transcripts (Relative Expression)
VEGFA Transcripts (Residuals)
c
CDKL1
(with cell-type-specific markers)
CDKL1 Transcripts (Relative Expression)
CDKL1 Transcripts (Residuals)
d
CDKL1
(without cell-type-specific markers)
CDKL1 Transcripts (Relative Expression)
CDKL1 Transcripts (Residuals)
e
EEF2K
(with cell-type-specific markers)
EEF2K Transcripts (Relative Expression)
EEF2K Transcripts (Residuals)
f
EEF2K
(without cell-type-specific markers)
EEF2K Transcripts (Relative Expression)
EEF2K Transcripts (Residuals)
g
SGSM3
(with cell-type-specific markers)
SGSM3 Transcripts (Relative Expression)
SGSM3 Transcripts (Residuals)
h
SGSM3
(without cell-type-specific markers)
SGSM3 Transcripts (Relative Expression)
SGSM3 Transcripts (Residuals)
